# Recent Progress in Recombinant Influenza Vaccine Development Toward Heterosubtypic Immune Response

**DOI:** 10.3389/fimmu.2022.878943

**Published:** 2022-05-19

**Authors:** Mark B. Carascal, Rance Derrick N. Pavon, Windell L. Rivera

**Affiliations:** ^1^ Pathogen-Host-Environment Interactions Research Laboratory, Institute of Biology, College of Science, University of the Philippines Diliman, Quezon City, Philippines; ^2^ Clinical and Translational Research Institute, The Medical City, Pasig City, Philippines

**Keywords:** heterosubtypic immunity, influenza, influenza antigen, recombinant vaccine, universal vaccine

## Abstract

Flu, a viral infection caused by the influenza virus, is still a global public health concern with potential to cause seasonal epidemics and pandemics. Vaccination is considered the most effective protective strategy against the infection. However, given the high plasticity of the virus and the suboptimal immunogenicity of existing influenza vaccines, scientists are moving toward the development of universal vaccines. An important property of universal vaccines is their ability to induce heterosubtypic immunity, i.e., a wide immune response coverage toward different influenza subtypes. With the increasing number of studies and mounting evidence on the safety and efficacy of recombinant influenza vaccines (RIVs), they have been proposed as promising platforms for the development of universal vaccines. This review highlights the current progress and advances in the development of RIVs in the context of heterosubtypic immunity induction toward universal vaccine production. In particular, this review discussed existing knowledge on influenza and vaccine development, current hemagglutinin-based RIVs in the market and in the pipeline, other potential vaccine targets for RIVs (neuraminidase, matrix 1 and 2, nucleoprotein, polymerase acidic, and basic 1 and 2 antigens), and deantigenization process. This review also provided discussion points and future perspectives in looking at RIVs as potential universal vaccine candidates for influenza.

## Introduction

Flu is an acute respiratory infection caused by influenza viruses. Influenza viruses have extensive genetic and antigenic diversity affecting pathogenicity, host range, and immune evasion, ultimately causing seasonal epidemics and pandemics worldwide. The World Health Organization (WHO) indicated that vaccination is the most effective prevention strategy against the infection (1). However, conventional vaccination may induce suboptimal immunogenicity and narrow the breadth of protection against the highly variable seasonal influenza strains. Therefore, influenza vaccination programs require constant monitoring and characterization of circulating viral strains for annual formulation. The emergence of COVID-19 pandemic in the early 2020 resulted in a decline in the overall reporting and positivity of influenza infection (2). As the 2021 to 2022 influenza seasons co-occurred with the pandemic, flu and COVID-19 co-infections have been reported and may have increased hospitalizations with significant mortality as reported in the United States of America (USA) (3). Concurrently, the COVID-19 pandemic may have affected the circulation of influenza virus in the community, potentially resulting to populations with decreased natural immunity against influenza viral strains or a decrease in viral antigenic drift (4). Alarmingly, the US Centers for Disease Control and Prevention (CDC) reported an increase in influenza A(H2N2)—a strain associated with higher hospitalization and deaths—in recent outbreaks in the USA, highlighting that influenza season preparedness should not be deprioritized (5). Several observational studies on patients, older populations and healthcare workers associated influenza vaccination with decreased COVID-19 infection rates, hospitalizations, morbidities, and mortalities, suggesting complementary protection by influenza vaccines against COVID-19 (6–11). A recent systematic review revealed that influenza vaccination increased in demand and acceptability among vulnerable populations during the COVID-19 pandemic (12). Clinical trials involving concomitant administration of trivalent and quadrivalent (including recombinant) influenza vaccines and COVID-19 mRNA vaccines have also showed no safety concerns or immune interference (13, 14). These observations prompted the CDC to recommend concurrent, sequential or in some cases, simultaneous administration of influenza vaccine with COVID-19 vaccine (15). Hence, despite the overall shift in the epidemiology of influenza infections at the time of the pandemic, research on influenza vaccination remains to be relevant.

Besides global monitoring programs for seasonal influenza, the WHO also pushes for initiatives on pandemic influenza preparedness, with vaccine development among its main components (16). However, with the current widespread use of egg-based methodologies in influenza vaccine production, recent estimates indicate insufficient immunization preparedness against another influenza pandemic (17). To circumvent these limitations, researchers worldwide studied other vaccine production strategies such as genetic recombination of immunogenic target genes. Advancements in recombination technologies and expression systems have facilitated the development of improved recombinant influenza vaccines (RIVs). RIVs are produced through expression of target proteins, genetic material, or virions in cell-based platforms. Unlike egg-based platforms, RIVs are considered safer, faster to produce, and more efficient in immune induction (18). RIVs have diversified into numerous types, including protein, DNA, virus-like particles (VLPs), and vector-based, which are potential universal vaccine candidates. Although there is no formal and globally accepted definition for universal vaccines, they should generally have at least 75% efficacy against symptomatic infections, provide heterosubtypic protection against all viral strains, induce durable and long-lasting immunity, and be safe for all ages (19, 20). Current global research focuses on finding vaccine candidates that can induce heterosubtypic immunity and protect individuals against different influenza subtypes (21). RIVs are being explored to induce heterosubtypic immunity, which points to their potential as universal vaccine candidates. A recent review discussed updates on universal RIV development based on targeting various influenza antigens (22). However, detailed discussion on vaccine candidates in the clinical and preclinical pipelines targeting both surface and internal antigens (i.e., polymerase complex proteins) using different RIV platforms to induce heterosubtypic immune response is still not available in the current literature.

The objective of this review paper was to highlight current progress and advancements in RIV development, focusing on the properties of the different influenza targets, current production platforms, and evidence of inducing heterosubtypic immunity. Specifically, this review discussed important concepts on influenza virus, infection, and vaccine development, and their implications in RIVs. While the focus of most influenza vaccine review articles is on HA-based vaccines, we highlighted in this review the other potential influenza targets and RIV production platforms to give a holistic overview on RIVs in the market and in the research pipeline. This review contributed to the growing knowledge on influenza vaccines through its detailed synthesis of existing and currently investigated RIVs targeting different viral proteins, including influenza mRNA vaccines.

## Influenza: The Virus and the Infection

### The Influenza Virus: Diversity, Genetics, and Proteins

Influenza viruses belong to *Orthomyxoviridae*, a family of pleomorphic enveloped virions with segmented, linear, single-stranded, negative-sense RNA genomes (23). There are currently four known genera of influenza viruses (A, B, C, D) with different host reservoirs and pathogenicity. Currently, influenza A is classified based on surface glycoproteins with 18 hemagglutinin (HA) and 11 neuraminidase (NA) subtypes, and influenza B with Yamagata and Victoria lineages (20, 24). Influenza C and D are classified into six (Taylor, Mississippi, Aichi, Yamagata, Kanagawa and Sao Paulo) and three (OK, 660 and Japanese lineages) lineages, respectively (25, 26). Although there are numerous subtypes, only influenza A can cause pandemics (e.g., H1N1 [“Spanish flu” of 1918], H2N2 [“Asian flu” of 1957], and H3N2 [“Hong Kong Flu” of 1968]) and annual epidemics (23).

Influenza viruses have broad antigenic variations mainly due to antigenic drift and shift. Antigenic drifts are minor but gradual changes brought by error-prone RNA-dependent RNA polymerase enzymes during genome replication which can incur pathogenic advantages such as immune evasion and decreased immunogenicity (20, 27). In contrast, antigenic shifts are more sudden, involve reassortment of antigenic genes producing novel influenza subtypes, and are often only observed among influenza A viruses due to their broad host distribution and co-occurrence with multiple strains (28–30). The CDC characterizes thousands of influenza viruses yearly to establish effective vaccine regimens against circulating strains (31). Vaccine efficacy for influenza typically falls between 50% to 70%, but often wanes from mismatches due to wrong predictions as well as antigenic drifts and shifts (32). Thus, developing universal influenza vaccines remains challenging.

**Figure 1:**
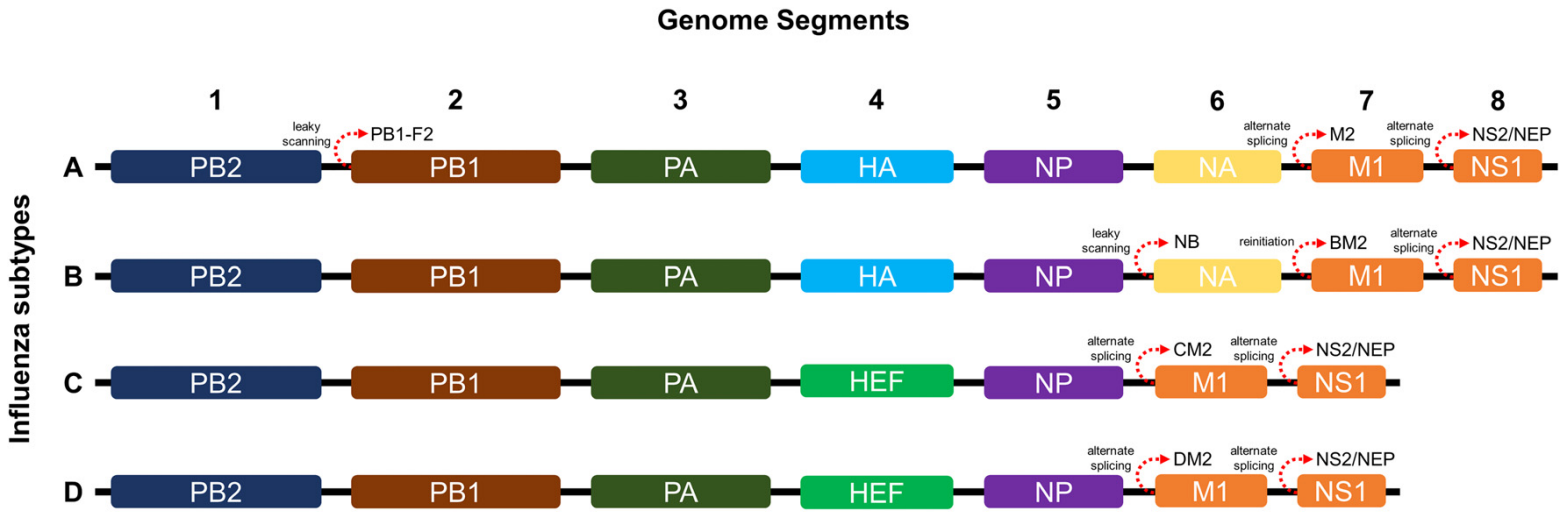
Genome segments and gene arrangements of influenza virus subtypes. The A and B subtypes contain eight segments, while the C and D subtypes contain only seven.

As shown in **Figure 1**, influenza A and B possess eight RNA gene segments, while C and D possess only seven (33). In influenza A, segments 1 and 2 encode polymerase basic proteins 1 and 2 (PB1 and PB2) respectively, 3 for polymerase acidic protein (PA), 4 for HA, 5 for nucleoprotein (NP), 6 for NA, 7 for matrix proteins (M1 and M2), and 8 for nonstructural proteins (NS1 and NS2) (34). Influenza B also has a similar arrangement (35), although M2 is replaced by NB in segment 6 and BM2 in segment 7 (36). Alternate reading frames also allow encoding of other proteins such as PB1-F2 of influenza A and BM2 of influenza B (37, 38). Influenza C and D share similar segments, except for HA and NA, which are replaced by hemagglutinin-esterase fusion protein (HEF) in segment 4, and M2 is found in segment 6 (39, 40). Influenza proteins differ in synthesis site and localization. HA, NA, HEF, M2, and NB are synthesized in membrane-bound ribosomes and become embedded within viral envelopes as transmembrane proteins, while PB1, PB2, PB1-F2, PA, NP, M1, NS1, and NS2 are synthesized in cytosolic ribosomes and while most are destined for nuclear localization, M1 and PB1-F2 localize underneath the viral envelope and to the mitochondria, respectively (33, 41–44).

HA is a homotrimeric surface glycoprotein involved in viral invasion, wherein each monomer consists of two subunits (HA1 and HA2) linked by disulfide bonds (45, 46). NA is a homotetrameric surface glycoprotein important for viral release and infection initiation (47). The glycosylation and stem length of these antigens affect influenza pathogenicity, transmission, emergence of new strains, and vaccine efficacy (39, 48, 49). M2 is a tetrameric protein generally functioning as an ion channel with differences across influenza genera (40). M1 is the most abundant protein in influenza viruses and is capable of oligomerizing and interact with NP-bound viral RNA segments or ribonucleoproteins (RNP) and cytoplasmic portions of HA and NA (50). PB1, PB2, and PA constitute the heterotrimeric RNA polymerase complex that associates with RNPs and forms nucleocapsids within virions that are transported to the host nucleus for viral gene transcription, synthesis, and expression (23, 28, 51). NP forms RNP complexes for viral genome transcription and replication (52). It is capable of self-oligomerization, which is crucial for its function (53) and is generally conserved and thus a viable vaccine target (54). Finally, NS1 is involved in viral genome expression and packaging, immune deregulation, inhibition of host genome expression, and pathogenesis (55), while NS2 exports viral RNPs to the cytoplasm (23).

### Influenza Infection: Pathogenesis and Immune Response

**Figure 2:**
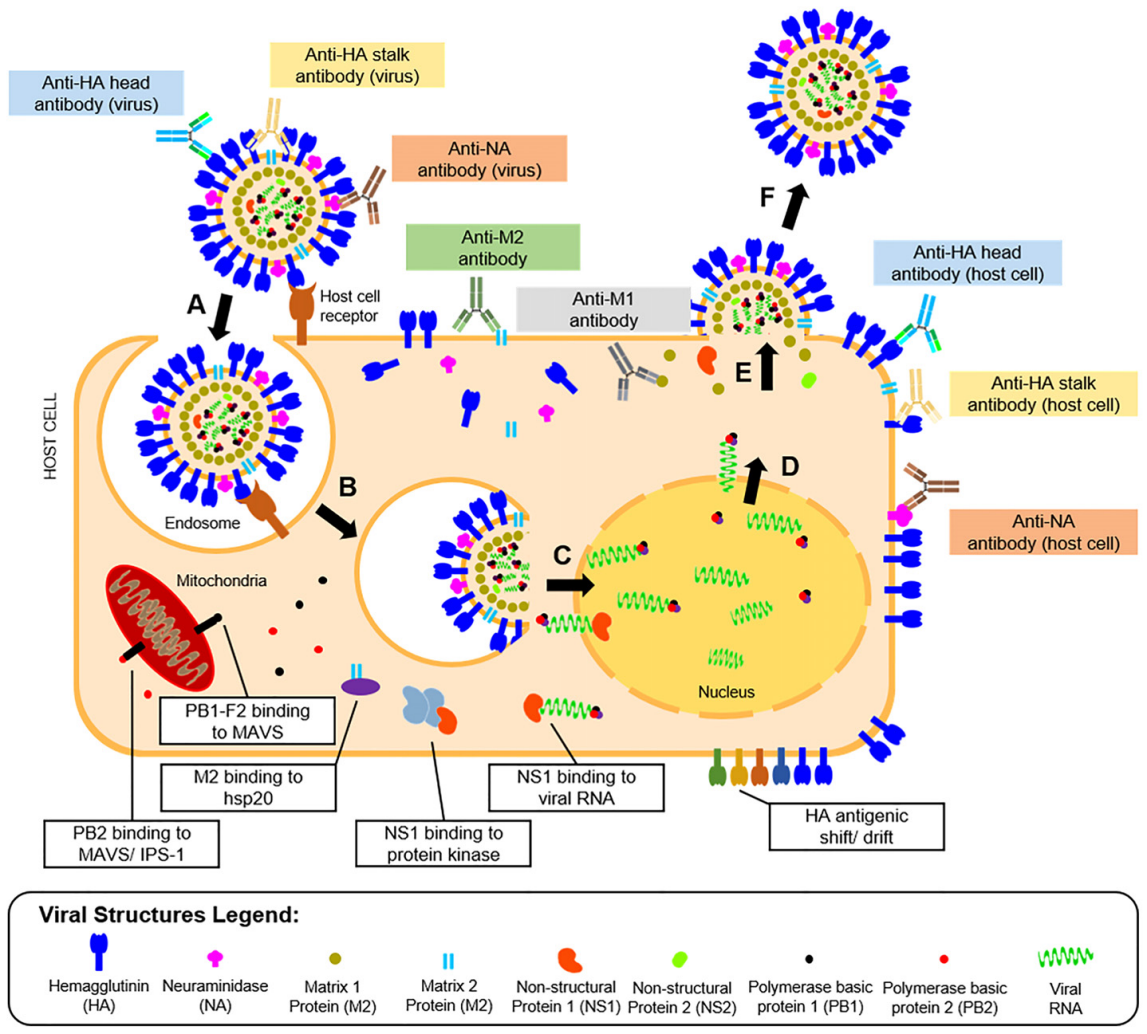
Life cycle, humoral immunity targets, and immune escape mechanisms of influenza viruses. The influenza infection starts with **(A)** the attachment of the viral HA protein to terminal sialic acid residues of host cell receptors on mucosal membranes. The process is followed by **(B)** endocytosis of the virus, where an endosome is formed inside host cells. The endosome is then acidified causing **(C)** fusion of the viral HA with the endosomal membrane and release of viral proteins and genome into the host cytoplasm. The viral genome is transported to the host cell nucleus, where it is **(D)** transcribed to messenger RNAs and eventually translated to viral proteins for progeny virions. **(E)** The viral proteins undergo post-translational modification at the host cell’s Golgi apparatus, eventually get packaged into progeny virions, and are then **(F)** released by budding from the host cell membrane where viral surface proteins are expressed. Influenza proteins expressed in different phases of the viral life cycle serve as targets for the humoral immune response (colored boxes). The most reported antibodies in influenza A are those targeting the HA head and stalk and the NA expressed in the released virions. However, other antibodies targeting the host cell-bound HA head and stalk, NA, M1, and M2 were also reported. The viral escape response from the host’s immune system was also described (white boxes), which include viral protein binding to cellular enzymes, and modification of viral protein antigens.

Vaccine development requires understanding of viral pathogenesis and host immune response. In humans, infection starts through binding of viral glycoproteins to sialic acid residues on epithelial cell surfaces of the upper and lower respiratory tracts (56) followed by influenza replication, as shown in **Figure 2**. Replication involves attachment of viruses to host cells, endocytosis, viral fusion, viral genome expression, virion assembly, budding, and release. Hutchinson (24) presented a detailed review of this process. Upon successful viral infection and replication within two to five days, patients can experience fever, colds, sore throat, congestion, chills, fatigue, vomiting, diarrhea, and abdominal pain (57). Symptoms of influenza infection are directly brought by the host’s immune response to the virus. On one hand (innate response), sensing of intracellular infection is accomplished by the detection of viral RNA through the cell’s toll-like receptors (TLR3,7) and retinoic acid inducible gene-I (RIG-I). This response causes the production of type I interferons and proinflammatory cytokines (58). Meanwhile, interaction of M2 with the NOD-like receptor family pyrin domain containing 3 promotes production of interleukins that aid in the adaptive immune response (59). Alveolar macrophages phagocytose infected cells, while natural killer cells recognize and lyse infected cells by interacting with cell-bound HA (60). Kreijtz et al. summarized the innate immune responses to influenza (61). On the other hand, the adaptive response involves production of antibodies specific to viral proteins on the virion or expressed on infected cells. Krammer described this response in detail (62). Antibody binding to HA globular heads on virions inhibits viral attachment to host cells, while binding to HA on the infected host cell surfaces mediates antibody-dependent cytotoxicity. Antibody binding to NA on virions inhibits the protein’s enzymatic activity limiting viral entry, while attachment to NA on infected cells activates complement-mediated cell lysis pathways. Aside from surface-expressed viral proteins, humoral immunity also targets internal viral structures such as M1, M2, and NP, which may be involved in different immune responses. Both cellular and humoral immune responses contribute to influenza protection in humans.

To combat the host’s immune responses, influenza viruses have escape mechanisms to innate and adaptive immunity, as presented in **Figure 2**. For instance, antigenic changes to the HA head help the virus escape humoral immunity or increases affinity of the protein to its host cell receptor (63). As will be highlighted later in this review, this adaptation is also a major hindrance to influenza vaccine development. Other viral proteins like M2 bind to heat shock proteins (HSP) (e.g., HSP40) to promote apoptosis of infected cells (64). Similarly, PB2 binds to mitochondrial antiviral signaling proteins to inhibit type I interferon production to promote cell apoptosis (65) or to interferon promoter stimulators to inhibit cytokine production (66). Lastly, binding of NS proteins to viral RNA and RNA-dependent protein kinase masks TLR recognition and RIG-I activity, limiting the innate immune response to the virus (67).

### Influenza Infection Epidemiology

Determination of global influenza burden remains challenging due to underreporting and lack of viral surveillance data worldwide. Before the COVID-19 pandemic, estimates indicate morbidity of about 50 million cases (68) and 650,000 deaths from influenza infections annually (69). Seasonal influenza outbreaks occur during winter in the Northern and Southern hemispheres (70). In the tropics and subtropics, outbreaks occur variably all year round, with significant rises during the second quarter (71). Most outbreaks last up to three months, but viral persistence mechanisms remain unknown. To circumvent the high incidence of influenza infection and mortality, and to prevent occurrence of another pandemic, the WHO created the Pandemic Influenza Risk Management Guidance, which highlighted the need for continuous development of vaccines and proper strategies for vaccine procurement, deployment, and prioritization among the population (16). As of 2019, researchers estimated production of more than 1.4 billion doses of influenza vaccines, scaling up to 8 billion doses, should a pandemic happen (17). For surveillance, the WHO created FluNet (https://www.who.int/tools/flunet/, accessed on March 18, 2022), a database of influenza cases and subtypes reported by national influenza centers and laboratories worldwide. Pre-pandemic, the circulating strains of influenza virus include influenza A(H1N1)pdm09, A (H3N2), B Victoria lineage and B Yamagata lineage.

At the onset of the COVID-19 pandemic, the number of specimens processed and reported worldwide for influenza significantly decreased by more than 99% (72). Concurrently, the positivity rate for influenza infection dropped from approximately 29% in early February 2020, to less than 0.1% in late May 2020. From May 2020 until June 2021, reports indicated influenza B Victoria lineage and influenza A H3N2 as the predominant global strains in circulation, and a potential extinction of influenza B Yamagata lineage (73). Influenza A(H1N1)pdm09 was sporadically reported. In late 2021 until early 2022, as the volume of specimens being tested for influenza virus begins to go back to its pre-pandemic rate, the positivity remains low at less than 6% (72). Influenza B Victoria lineage and influenza A H3N2 remained to be the predominant strains. These changes in the epidemiological dynamics of the influenza virus during the pandemic may have direct impact in the development of vaccines in the future. Some possibilities include focusing on the vaccine production without influenza B Yamagata lineage representation or increasing the influenza A (H3N2) antigenic targets to two in quadrivalent vaccines (73).

## Evolution of Influenza Vaccines

### History of Influenza Vaccines

**Figure 3:**
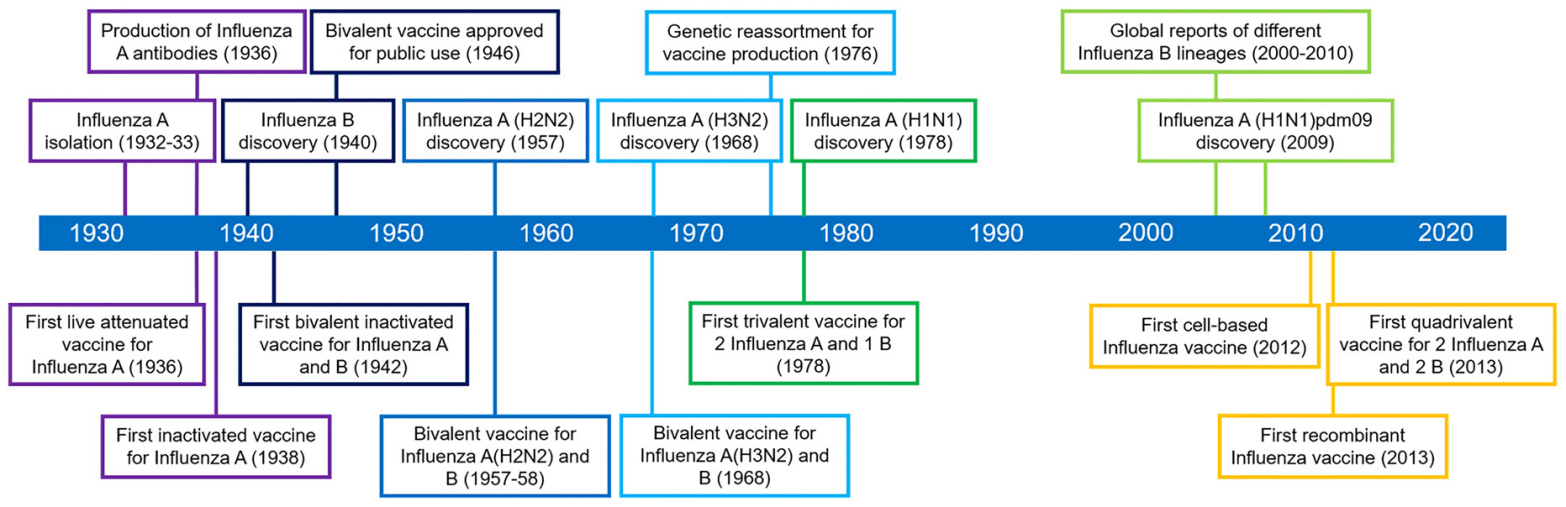
Historical timeline of influenza vaccine development.

Influenza epidemic outbreaks have been reported from the 1500s until the 1900s worldwide. Among these outbreaks, historians dubbed the Spanish influenza pandemic of 1918–1919 as the worst medical holocaust in history (74). Influenza vaccine development (**Figure 3**) began only after the pandemic (1932), when scientists isolated influenza A from infected patients’ nasal secretions (75). In subsequent years, scientists successfully transmitted the virus to mice and embryonated chicken eggs. In 1936, independent researchers produced the first neutralizing antibodies against the virus (76). In the same year, the first live attenuated influenza A vaccine has been attempted in the Union of Soviet Socialist Republics (USSR) using an egg-based production platform (77). Concurrently, scientists from the USA utilized formalin-inactivated virus [Influenza A/PR8 (H1N1)] isolated and purified from the allantoic fluid of chick embryos to produce the first inactivated influenza virus vaccine (78). These milestones highlight the development of monovalent vaccine against influenza A. In 1940, an antigenically distinct influenza B virus was discovered (79). In response to this discovery, the first bivalent inactivated vaccine targeting both influenza A and B was produced based on the previous production protocols, but using half of allantoic fluids with influenza A/PR8 (H1N1) and half with influenza B/Lee lineage (80). In 1945-1946, the vaccine was licensed for public use. A few years later, influenza A(H2N2) was discovered and resulted in a pandemic, replacing the previous influenza A strain in the circulation. In response, a bivalent inactivated vaccine targeting influenza A(H2N2) and B was created. After another decade, the target strain for the formulation was replaced with influenza A(H3N2) and B in response to the new strain in circulation (80). In 1978, influenza A(H1N1), an analog of the influenza A(H2N1), was discovered co-circulating with A(H3N2). This situation paved the way for the development of trivalent inactivated vaccines targeting the two co-circulating influenza A and one influenza B (79). In 2009, a new influenza A strain, A(H1N1)pdm09, replaced the circulating A(H1N1) and caused another pandemic, leading to a change in the influenza A(H1N1) target in vaccine formulation (80). From the early 2000’s to 2010, different influenza B virus lineage (predominantly B/Yamagata and B/Victoria) have been reported from different parts of the world, with predominance of one lineage among the other in certain regions. Since only one influenza B target is included in the trivalent vaccine formulation, variabilities in vaccine efficacies have been reported (80). Hence, in 2013, WHO recommended a quadrivalent vaccine formulation for seasonal influenza, to include two influenza A and B targets (81). For the years 2021 to 2022, both US Food and Drug Administration (US FDA) and the WHO recommended the formulation of trivalent egg-based vaccines to include influenza A/Victoria/2570/2019 (H1N1) pdm09-, influenza A/Cambodia/e0826360/2020 (H3N2)-, and influenza B/Washington/02/2019 (Victoria lineage)-like viruses. For trivalent cell-based or recombinant vaccines, the formulation should include influenza A/Wisconsin/588/2019 (H1N1) pdm09-, influenza A/Cambodia/e0826360/2020 (H3N2)-, and influenza B/Washington/02/2019 (Victoria lineage)-like viruses (82, 83). For both egg- and cell-based quadrivalent vaccines, the influenza B/Phuket/3073/2013-like virus is recommended for inclusion. In summary, the epidemiologic characteristics and prevalence of influenza subtypes reported globally dictate the direction of future vaccination strategies against the infection.

For most of its history, influenza vaccines were produced using eggs, which are low-throughput and cannot be administered to individuals with egg allergy. Advancement in vaccine production led to the development of cell-based influenza vaccine platforms and recombinant vaccines. Initially, in 1976, researchers introduced genetic reassortment as a method for faster production of vaccines. In this method, plasmids containing selected genes from different influenza virus targets are expressed in cell lines (84). The resulting reassortant is an attenuated, biologically active virus strongly expressing the viral antigen. From the initial reassortment method, the principle of recombination has been considered to produce RIVs that may target specific influenza antigens without the need to produce biologically active virions. Ultimately, recombinant vaccines can be used to overcome limitations of traditional vaccine production and pave the way to target more conserved and cross-reactive antigens toward heterosubtypic immunity (85). RIVs are produced in egg-free environments and are faster to manufacture, indicating ideal use in global emergencies such as during pandemics (86). In 2012, a vaccine from a cell-cultured influenza virus was introduced (79). Meanwhile, the first RIV was approved by the US FDA in 2013 (87). In the recent years, two additional RIVs have been approved in the global market for use in seasonal flu immunization.

### Types of Influenza Vaccines

Influenza vaccines are categorized based on their production platforms (i.e., inactivated, live, recombinant) and expression types (whole virus, split-virus, subunit). **Table 1** summarizes the characteristics of licensed influenza vaccines worldwide. The WHO vaccine safety basics manual (88) and Sekiya et al. (89) thoroughly discussed the properties and differences between the different vaccine types. Inactivated WVVs use whole viruses in immunization and may induce strong immune responses. Meanwhile, inactivated SVVs and subunit vaccines are composed of disrupted viruses or specific viral components, respectively, and are less immunogenic. Repeated immunization and/or addition of adjuvants to these vaccines are usually employed to improve their immunogenicity. Live attenuated virus vaccines (LAVs) use weakened, non-replicating viruses that may imitate an actual influenza infection, thereby inducing more cellular and humoral immune responses. Although the viruses used in these vaccines are noninfectious, strong caution is still necessary when used in immunocompromised populations or those with underdeveloped immune systems. Lastly, RIVs are produced by recombination and expression of viral proteins or virions in vectors propagated in cell lines. The properties of RIVs are highlighted in the following sections of this review.

**Table 1:** General characteristics and notable examples of influenza vaccines licensed for use against seasonal or pandemic influenza, categorized based on their type and production platform.

**Vaccine Type**	**Production Platform (Notable Vaccines)**	**Limitations^1^**
Inactivated, whole virus	Egg-based (e.g., Daronrix^®^, 3Fluart^®^)	- May have strong immunogenicity, but may cause infection symptoms - Not suitable for people with egg allergies (for the egg-based vaccines)
	Cell-based (e.g., Celvapan^®^, Vepacel^®^)	
Inactivated, split virus	Egg-based (e.g., AdimFlu^®^, Afluria^®^, Arepanrix^®^, Fluarix^®^, FluLaval^®^, Fluzone^®^)	- May induce only moderate immune responses in previously vaccinated or infected individuals - Requires frequent updating of target strains due to the highly specific nature of the immune response - Not suitable for people with allergies to egg (for the egg-based vaccines)
	Cell-based (e.g., Preflucel^®^)	
Inactivated, subunit virus	Egg-based (e.g., Agrippal^®^, Fluvirin^®^, Influvac^®^)	- May have low immunogenicity - May require adjuvants to increase immunogenicity - May not form immunological memory from the antigen - Not suitable for people with allergies to egg (for the egg-based vaccines)
	Cell-based (e.g., Celtura^®^, Flucelvax^®^, Grippol^®^)	
Live, attenuated virus	Egg-based (e.g., CAIV-T^®^, Fluenz^®^, FluInsure^®^, Flumist^®^, Nasovac^®^, Ultravac^®^)	- May induce harm to immunocompromised populations or those with underdeveloped immune systems - More susceptible to immunization errors, contamination, and reversion to pathogenic form - Not suitable for people with allergies to egg (for the egg-based vaccines)
Recombinant	Cell-based (e.g., Cadiflu-S^®^, FluBlok^®^, Supemtek^®^)	- Production methods may be costly and still under investigation - Not suitable for children and people not primed with previous infection

^1^Based on the WHO (88) and Sekiya et al. (89).

Most seasonal vaccines are multivalent, eliciting immune responses against specific target variants of both influenza A and B subtypes. Pandemic vaccines remain monovalent (with the exception of the trivalent Adimflu-S^®^) and target specific viral strains associated with previous pandemics (17). Despite advancements in vaccine production, the development of universal influenza vaccines remains slow (17). Recently, the CDC described recombinant vaccine production as potentially faster, more effective, and comparably safe (i.e., not induce allergic reactions) (18). Supporting this, Buckland et al. described the scale-up process for producing recombinant vaccines to be as short as 38 days following current Good Manufacturing Practices, compared with more than 20 weeks for other traditional vaccines (90). In addition, a recently published clinical trial suggested that recombinant vaccines induce a more robust humoral immune response compared with other commercially available cell- and egg-based vaccines (91). The next section highlights the current progress in RIV development, with detailed focus on the existing vaccines in the market and in the pipeline.

## Recombinant Influenza Vaccines

### Recombinant Vaccine Technology

**Figure 4:**
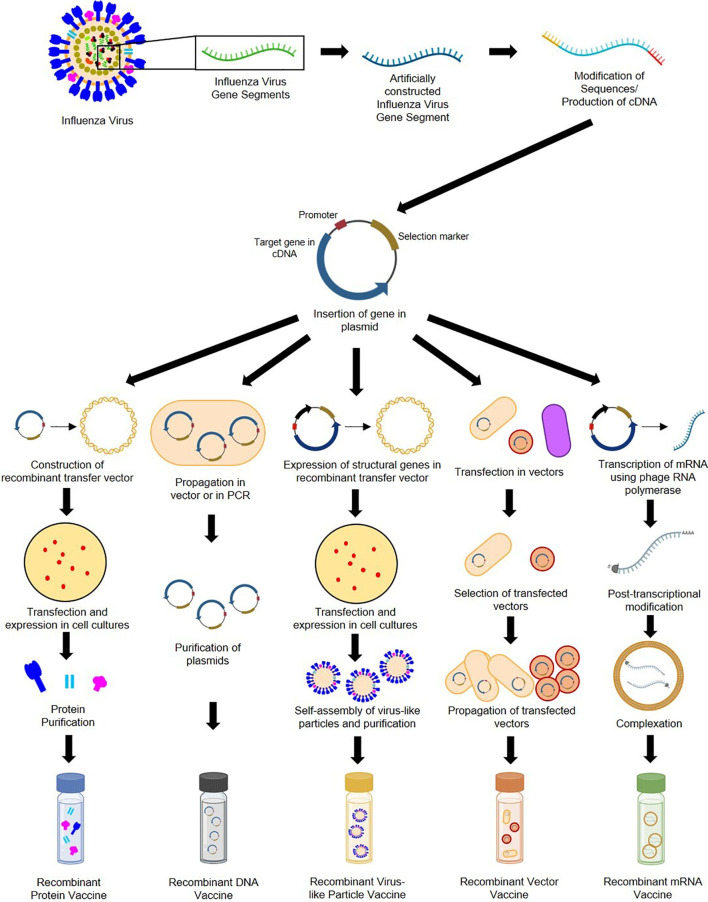
General construction process of recombinant protein, DNA, VLP, vector-based, and mRNA influenza vaccines.

Genetic recombination is the rearrangement of DNA sequences of organisms often occurring naturally producing mutations and subsequent population diversity across species or involved in DNA repair mechanisms (92). Recombination can also be introduced artificially as observed in 1972 when Paul Berg and his team became the first to construct recombinant DNA by inserting segments of lambda phage genes and the galactose operon of *Escherichia coli* into simian virus 40 DNA (93). Recombination provides solutions in food production, pharmaceuticals, diagnostics, therapeutics, biofuel, bioremediation, and, more recently, vaccine development (94). As seen in **Figure 4**, recombinant vaccine development paved the way for the production of subunit (e.g., protein, carbohydrate), conjugate, live recombinant vector (bacterial, viral), DNA, VLP (95), and more recently, mRNA vaccines (96).

Recombinant subunit vaccines contain defined non-replicating and noninfectious antigenic components of pathogens (97). They can be based on peptides, polysaccharides, nucleic acids, etc., which can elicit different immune responses (95). Construction of protein-based RIVs begins with sequences of target influenza antigens from various subtypes (which can be modified during the recombination process). These sequences are generated into complementary DNA and inserted into plasmids containing an origin of replication, promoter, and selection markers (98). Plasmids are then placed in transfer vectors (e.g., baculovirus DNA) before transfection in insect, mammalian, yeast, or bacterial cell cultures to allow expression of protein antigens (97, 99–101). Antigens expressed by eukaryotic cells can undergo post-translational modifications and mimic natural infections but without disease outcomes (98). Recombinant proteins produced are then purified prior to immunization. DNA-based RIVs are also subunit vaccines and are safer than LAVs (102). Construction of DNA-based RIVs also requires plasmids containing influenza antigens, which are propagated in cloning vectors or through PCR, followed by purification prior to administration (103, 104). Their delivery may require methods such as electroporation (105), gene guns (98), microneedle arrays (106) and cell-penetrating peptides (107). They are also vulnerable to enzymatic attacks, which can be mitigated by liposome encapsulation or dendrimer binding (95). VLP-based RIVs are also subunit vaccines but mimic virion arrangements wherein antigens are encased in structural viral proteins with or without an envelope (95). They are most often produced using baculovirus vectors in insect-cell expression systems (108). VLPs require the inclusion of influenza structural genes together with the antigenic targets and self-assembly of VLP structures prior to collection and purification (109). VLPs require extensive downstream processing and purification to ensure safety, stability, potency, and consistency, which are potential production bottlenecks (110, 111). Finally, vector-based RIVs are considered as the most immunogenic, eliciting humoral, cellular, mucosal, or systemic immune responses (112). While LAV risk reversion to wild-type and increase in virulence, recombinant vectors are safer, but may undergo pre-host (during manufacture) and within-host evolution, losing inserted heterologous antigens and having decreased immunogenicity (113). Construction of recombinant vectors requires transfection of plasmids carrying influenza antigenic targets into vectors (can be viral [e.g., modified vaccinia virus Ankara [MVA], adenovirus, norovirus] or bacteria [e.g., *Lactococcus*, *Salmonella*]), and expression in propagating cells before purification (114–118).

With the advent of nucleic acid-based vaccine development against COVID-19, major vaccine companies are now also moving toward influenza mRNA vaccine production. In the context of COVID-19, Yadav et al. classified mRNA vaccines as recombinant (96). Hence, in this review, we will also tackle some current advances in influenza mRNA vaccine development. Generally, mRNA vaccine is produced by *in vitro* transcription of mRNA from a DNA sequence corresponding to the target viral protein. The resulting mRNA product typically resembles mature eukaryotic mRNA (i.e., with 5’ cap, flanking untranslated regions, open reading frame for the protein, and poly(A) tail) that can be readily translated by the host’s translational machinery (119). Unlike other recombination platforms that still rely on cell-based expression of the proteins or nucleic acids, the entire process of manufacturing mRNA can be done synthetically in production systems, making the process easier, faster and with high fidelity (120). As with DNA-based vaccines, mRNA vaccines typically require effective delivery methods to induce immune reaction. These methods include electroporation, complexation with cations (peptides, nanoemulsions, polyethylene glycol-lipid, polyethylenimine polymer, polysaccahrides), or delivery in lipid nanoparticles (119, 121).

### HA-Based Recombinant Vaccines

#### HA Head-Based Recombinant Vaccines

Currently, only HA-based RIVs have been approved for use by global regulatory bodies. Aside from being the most dominant surface protein in influenza viruses, HA is also beneficial in vaccine development because of its inherent immunogenicity and bulky configuration, making it readily accessible to neutralizing antibodies (122). The HA head is immunodominant, conferring a strain-specific immune response to vaccinated hosts (62). Antibodies produced against the HA head have variable activity against different HA subtypes, as summarized by Hashem (123). Typically, antibodies produced from HA heads exhibit hemagglutination inhibition activity (124); this reaction has become a major technique in evaluating vaccine efficacy. Other common antibody responses to the HA head include prevention of viral attachment or receptor binding (125, 126), repression of viral release (126, 127), and increased avidity (128). However, a major downside of HA is its inherent plasticity due to antigenic drift (129). This makes antibodies produced from HA-based vaccines specific to only certain subtypes of influenza.

Flublok^®^ was the first HA-based RIV approved in the USA, manufactured by Sanofi Pasteur (France). Flublok^®^ is a quadrivalent vaccine recommended for administration among 18–49-year-old populations (130). It is produced through the insertion of HA genes into the *Autographa californica* nuclear polyhedrosis virus, a baculovirus vector, which is used to infect the *express*SF+^®^ insect cell line for mass production (131). Phase II clinical trials indicated 135 µg as the vaccine’s ideal dose, with generally accepted safety profiles among vaccinated populations (132, 133). In a phase III placebo-controlled trial, Flublok^®^ was found to produce high levels of HA antibodies among vaccinated individuals of 18–49 years of age (134). Meanwhile, in two phase III trials with Fluzone^®^ comparator, researchers found that Flublok^®^ produced non-inferior HA antibody titers among the elderly (65 years old and above), with seroconversion favoring HA proteins of influenza A over B (135, 136). A separate phase III trial added that among 18–49-year-old vaccinated populations, high seroconversion rates against recombinant HA proteins from influenza A and B (Yamagata lineage), but not influenza B (Victoria lineage), can be observed (137). Researchers also found that Flublok^®^ induced a superior CD4^+^ T-cell response compared with other commercial split-virus and subunit vaccines, presenting initial evidence of cellular immune response elicited by the RIV (138). With promising findings on its immunogenicity and safety, Flublok^®^ received its first approval from the US FDA for its trivalent formulation in January 2013 (139) and its quadrivalent form in October 2016 (140).

Sanofi Pasteur also manufactures Supemtek^®^, the only HA-based RIV approved by the European Medicines Agency (EMA) for administration to adult patients (141). Supemtek^®^ has the same formulation and production process as Flublok^®^; hence, clinical trials supporting the efficacy and safety of Flublok^®^ are the same for Supemtek^®^. Both Flublok^®^ and Supemtek^®^ were indicated as safe for populations with egg allergies (130, 141). Meanwhile, in India, CPL Biologicals manufactured the country’s first recombinant VLP influenza vaccine called Cadiflu-S^®^, first approved by the Drugs Controller General of India in November 2016 (142). Cadiflu-S^®^ is produced through expression of recombinant HA, NA, and M1 influenza proteins using a non-specified baculovirus vector in Sf9 insect cell lines (142, 143). Although there is no published literature on its efficacy and safety, a documented phase III trial concluded that it induced acceptable HA antibody titers in patients, with a more than 70% seroconversion rate (143). Besides Flublok^®^, Supemtek^®^, and Cadiflu-S^®^, other HA-based recombinant vaccines are now in the global vaccine pipeline undergoing clinical trials (**Table 2**).

**Table 2:** Clinical trials for HA-based recombinant vaccines registered in clinicaltrials.gov for the last decade as of April 14, 2022.

Clinical Trial Identifier	Vaccine Description	Trial Phase*	Trial Site, Status (Sponsor/Manufacturer)
**Recombinant HA**			
NCT03450915	Recombinant vaccine with nine multimeric conserved antigenic sites from influenza A and B (M-001)	Phase III	Poland, Completed- 2021 (BiondVax Pharmaceuticals)
NCT01767896	Recombinant cell culture-derived HA vaccine against seasonal influenza (ASP7374)	Phase III	Japan, Completed- 2013 (UMN Pharma)
NCT01195038	Recombinant H5N1 vaccine, booster shot	Phase II	Japan, Completed- 2011 (UMN Pharma)
NCT03283319	Recombinant HA vaccine based on full-length H7 (PanBlok H7)	Phase II	Australia, Completed- 2020 (Vaxine Pty)
NCT00966238	Recombinant fusion protein with *Salmonella typhimurium* flagellin, TLR ligand fused to HA head based on influenza A H1 Solomon Islands (VAX125)	Phase II	USA, Completed- 2014 (VacInnate Corp)
NCT01450579	Recombinant HA-based vaccine (ASP7373)	Phase II	Japan, Completed- 2017 (UMN Pharma)
NCT03814720	Recombinant HA stem-based vaccine with ferritin protein from *Helicobacter pylori* (VRCFLUNPF099-00-VP)	Phase I	USA, Completed- 2021 (NIAID)^1^
NCT04451954	Adjuvanted and non-adjuvanted recombinant influenza vaccine with two strains of H3 and 2018–2019 NH H3 strain	Phase I	USA, Completed- 2021 (Sanofi Pasteur)
NCT02015494	Quadrivalent recombinant HA-based vaccine (VAX2012Q)	Phase I	USA, Unknown (VaxInnate Corp)
NCT02206464	Recombinant DNA plasmid based on H7 (VRC-FLUDNA071-00-VP)	Phase I	USA, Completed- 2019 (NIAID)
NCT02335164	Recombinant H5 vaccine (AdVax)	Phase I	Australia, Completed- 2019 (Vaxine Pty)
NCT03789539	Recombinant vaccine with HBc-4M2eh (Uniflu)	Phase I	Russia, Unknown (VA Pharma)
NCT00776711	Recombinant DNA plasmid based on H5 (VRC-AViDNA036-00-VP)	Phase I	USA, Completed- 2017 (NIAID)
NCT01172054	Inactivated vaccine with recombinant H1N1 (VAX128)	Phase I	USA, Completed- 2012 (VaxInnate Corp)
NCT01250795	Recombinant HA vaccine based on H5N1 (influenza A/Indonesia/05/2005) (HAI-05)	Phase I	USA, Completed- 2016 (Fraunhofer)
NCT01560793	Recombinant inactivated subunit vaccine based on H5N1 (VAX161B)	Phase I	USA, Completed- 2014 (VacInnate Corp)
NCT01658800	Recombinant vaccine based on H5N1 (VAX161C)	Phase I	USA, Completed- 2015 (VacInnate Corp)
NCT01177202	Recombinant fusion protein vaccine based on influenza A/California/04/09 (H1N1) (HAC1)	Phase I	USA, Completed- 2016 (Farunhofer)
NCT00858611	Recombinant DNA trivalent vaccine, prime-boost (VRC-FLUDNA047-00-VP)	Phase I	USA, Completed- 2017 (NIAID)
**Recombinant VLP**			
NCT04120194	Recombinant quadrivalent nanoparticle influenza vaccine composed of four influenza strains from 2019–2020 Northern Hemisphere influenza season adjuvanted with Matrix-M (NanoFlu)	Phase III	USA, Ongoing (Novavax)
NCT03301051	Quadrivalent, plant-derived virus-like particle vaccine composed of H1, H3, and two B HA proteins	Phase III	USA, Completed- 2020 (Medicago)
NCT04034290	Recombinant pseudo-adenoviral vaccine based on influenza A (GamFluVac)	Phase II	Russia, Completed- 2020 (GRIEM)^2^
NCT02918006	Recombinant adenovirus vector vaccine expressing HA adjuvanted with TLR3 (VXA-A1.1)	Phase II	USA, Completed- 2018 (Vaxart)
NCT04622592	Adjuvanted quadrivalent virus-like particle vaccine composed of H1, H3, and two B HA proteins	Phase I/II	USA, Ongoing (Medicago)
**Reassortant LAV**			
NCT00853255	Live attenuated virus with avian influenza H7N3 (6–2) AA ca (A/chicken/British Columbia/CN-6/2004 × A/Ann Arbor/6/60 cold adapted)	Phase I	USA, Completed- 2013 (NIAID)
NCT01175122	Live attenuated recombinant vaccine based on H2N3 (6–2) AA ca (A/Swine/Missouri/4296424/2006 (H2N3) × A/Ann Arbor/6/60 cold adapted)	Phase I	USA, Completed- 2013 (NIAID)
NCT02957656	Live attenuated recombinant vaccine based on H7N9 (6–2) AA ca (A/Anhui/1/2013 (H7N9) × A/Ann Arbor/6/60 cold adapted)	Phase I	USA, Completed- 2018 (NIAID)
NCT00922259	Live attenuated recombinant vaccine based on H7N7 (6–2) AA ca (A/Netherlands/219/03 (H7N7) × A/Ann Arbor/6/60 cold adapted)	Phase I	USA, Completed- 2013 (NIAID)
NCT04650971	Recombinant attenuated vaccine (nasal/aerosol) based on influenza A/H1N1pdm09 virus (UniFluVec)	Phase I	Russia, Completed- 2020 (Pharmenterprises Biotech)
NCT01006798	Recombinant live, competent adenovirus type 4 with H5N1 influenza Vietnam 1194 HA (Ad4-H5-Vtn)	Phase I	USA, Completed- 2020 (Emergent Biosolutions)
NCT03300050	Recombinant LAV with chimeric H8 head, H1 stalk and N1 (cH8/1N1 LAIV)	Phase I	USA, Completed- 2021 (The Emmes Company/GlaxoSmithKlein)
NCT03553940	Live intranasal vaccine with H3N2 M2SR (A/H3N2/Bris 10)	Phase I	USA, Completed- 2021 (NIAID)
**Recombinant mRNA**			
NCT04956575	mRNA-1010, quadrivalent, HA/NA-based (A/H1N1, H3N1, B/Yamagata and B/Victoria)	Phase I/II	USA, Ongoing (ModernaTX)
Unregistered	mRNA-5400/5401, monovalent, HA-based (A/H3N2), complexed with lipid nanoparticles	Phase I	USA, Unknown (Sanofi/Translate Bio)
NCT05052697	PF-07252220, bivalent, HA-based (A/H1N1, B/Yamagata)	Phase I	USA, Ongoing (Pfizer/BioNTech)
NCT03076385	VAL-506440, monovalent, HA-based (A/H10N8)	Phase I	USA, Completed- 2018 (ModernaTX)
NCT03345043	VAL-339851, monovalent, HA-based (A/H7N9)	Phase I	USA, Completed- 2021 (ModernaTX)

*For vaccines with multiple trials, only the latest phase trials are presented.

^1^National Institute of Allergy and Infectious Diseases, USA.

^2^Gamaleya Research Institute of Epidemiology and Microbiology, Russian Federation.

Panblok is a vaccine composed of full-length HA from influenza A H7 manufactured by Vaxine Pty (Australia). Similar to Flublok^®^ and Supemtek^®^, Panblok is produced in *express*SF+^®^ insect cell line infected with HA-expressing *Autographa californica* nuclear polyhedrosis virus (144). In its phase I/II clinical trial, vaccination with an oil-in-water glucopyranosyl adjuvant induced acceptable seroconversion in populations aged less than 65 years, with only mild adverse effects (144). In a more recent phase II clinical trial (NCT03283319), Panblok was adjuvanted with AS03 (α-tocopherol-, polysorbate-80-, and squalene-based) or MF59 (squalene-based). No results have been published yet for this vaccine, but regular updates are being uploaded (clinicaltrials.gov, accessed on November 10, 2021). The use of adjuvants was considered to increase the immunogenicity of H7, known to induce a weak response (145).

M-001 is a multimeric, recombinant, 50-kDa polypeptide vaccine with nine conserved HA, NP, and M1 epitopes reactive to B and T cells conferring both cellular and humoral immunity. M-001 was primarily produced in *E. coli via* fermentation and subsequent purification (146). In 2014, manufacturers claim that M-001 production takes only 6–8 weeks, and thus it can meet demands, especially in influenza pandemics (147). Developed by BiondVax Pharmaceuticals (Israel) as a universal influenza vaccine, it recently completed its phase III clinical trial. In its initial clinical trial phases, researchers found M-001 to induce acceptable immune response and safety profiles among the elderly (147). Its recently concluded phase II clinical trial (NCT03058692) showed significant differences in CD4^+^ and CD8^+^ T-cell responses in the M-001 plus seasonal vaccine group compared with the placebo plus seasonal vaccine group. Meanwhile, its phase III clinical trial (NCT03450915) indicated significant protection against influenza in two different seasons, with an acceptable safety profile. No formal publications for the phase II and III clinical trials are available yet, but their results can be viewed in clinicaltrials.gov (clinicaltrials.gov, accessed on November 10, 2021). The phase III clinical trial for M-001 was declared unsuccessful due to its failure to meet the target endpoints (https://www.biondvax.com/clinical-trials, accessed on March 23, 2022).

VAX125 is an innovative recombinant protein fusing flagellin type 2 from *Salmonella* and a TLR5 ligand to the globular HA head of influenza A H1N1 (148). Similar to M-001, this vaccine was produced through bacterial fermentation, which is more efficient and inexpensive compared with traditional expression systems (148). In its preliminary phase clinical trial, researchers found that VAX125 is generally safe among populations aged 18–49 years old, producing high immunogenic reactions at 1–3-µg doses (149). In a follow-up phase II trial, researchers indicated that a 5-µg dose is also safe and effective in geriatric populations (65 years old and above), inducing up to a 12-fold increase in HA antibodies (148). Production and testing of VAX-125 has already been discontinued (https://adisinsight.springer.com/drugs/800029053, accessed on March 23, 2022) while there are currently no insights on the status of other vaccine candidates, such as VAX2012Q, VAX128, VAX161B, VAX161C, from its manufacturer (VaxInnate Corp). Meanwhile, AS7374 and AS7373 are recombinant HA vaccines in their late clinical trial stages that target seasonal and pandemic influenza, respectively. Unfortunately, their manufacturer, UMN Pharma, and its partner company terminated their vaccination agreement, resulting in the withdrawal of marketing approval application (i.e. last step before releasing the vaccine to the market) for AS7374 and discontinuation of AS7373 production (150).

Another promising recombinant HA-based vaccine is Nanoflu, produced by Novavax Inc. (USA). Nanoflu is a quadrivalent nanoparticle-based vaccine with a proprietary Matrix-M™ adjuvant. It is produced by high-level expression of HA in *Spodoptera frugiperda* insect cells (Sf9) using the *A. californica* nuclear polyhedrosis virus as the expression vector (151). The resulting purified vaccine is then combined with Matrix-M™. Matrix M™ is derived from *Quillaja* plants and composed of saponin with cholesterol and phosphatidylcholine arranged into a cage-like nanoparticle. The combination was previously shown to activate innate immune cells and enhance antigen presentation and delivery (152). In the vaccine’s phase I/II clinical trial (trivalent form), researchers found similar safety and greater humoral immune response at a 60 µg dose of Nanoflu compared with another high-dose commercial vaccine (153). In their recently published phase II clinical trial, Shinde et al. found a significant increase in humoral and cellular homologous immune responses induced by Nanoflu compared to another commercial high-dose inactivated vaccine. They also found acceptable tolerance to adjuvanted and non-adjuvanted forms of Nanoflu (154). A phase III clinical trial also reported mild to moderate adverse reactions from the vaccine (155). Portnoff et al. found that the vaccine induced production of broadly neutralizing antibodies against conserved regions in the HA head (specifically H3), indicating potential heterosubtypic immunity (156). Overall, the vaccine trials are considered successful and Nanoflu is expected to undergo late-stage clinical trials (https://ir.novavax.com, accessed on March 23, 2022).

Another quadrivalent VLP RIV is the plant-derived MT-2271 produced by Medicago, Canada. MT-2271 contains HA of influenza A H1 and H3, and two influenza B subtypes. Vaccine production relies on the expression of influenza HA genes in *Agrobacterium tumefaciens* infected with 2X35S/CPMV-*HT* expression vector (157). The vaccine is then mass-produced in vacuum-infiltrated *Nicotiana benthamiana via* transient expression (158). Unlike traditional animal cell-based vaccine production, D’Aoust and colleagues described this plant-based method to be more efficacious (producing high antigenic property), high yield (up to 1,500 doses per kilogram of infiltrated leaves), and highly scalable for pandemic response (vaccine can be produced within three weeks after the release of viral genetic sequences) (159). In a phase III clinical trial, researchers reported that a 30 µg vaccine dose is safe, induced homologous and heterologous humoral and cellular immune responses in 18–49-year-old populations, and provided a lower but consistent response in populations above 50 years old (160). Its phase III clinical trial was completed in 2020; Ward et al. described the vaccine to be safe and to induce significant homologous humoral response with acceptable lot-to-lot consistency for all viral strains used (161). Despite these results, the trial was not able to achieve its primary endpoint for adult population, and Medicago is still re-evaluating the licensing of the vaccine (https://www.mt-pharma.co.jp/e/news/assets/pdf/e_MTPC200428.pdf, accessed on March 23, 2022).

GamFluVac is a pseudo-adenoviral RIV produced *via* a method patented by NT Pharma (China). Non-replicating nanoparticles are created using the human adenovirus type 5 vector expressing HA from both influenza A and B and mixed with an immunostimulating acidic peptidoglycan (162). The vaccine is then administered intranasally. Adenovirus was used given its strong immunogenicity, ability to penetrate the human mucosal epithelia (intranasal route), high propagation yield, stability (can be lyophilized and refrigerated), noninfectivity, and manipulability in order to create recombinant forms (163). One phase I and phase II clinical trials related to this vaccine are completed (NCT03651544, NCT04034290; clinicaltrials.gov, accessed on November 10, 2021). Unfortunately, no published results have been released yet. The current status of production and licensing of GamFluVac is not declared in the literature.

VXA-A1.1 is an oral flu vaccine developed by Vaxart, USA. It is produced by expressing the viral proteins in HEK293 cells through a BJ5183-AD1 bacterial vector (164). The resulting recombinant protein is adjuvanted with a TLR3 agonist (165). In a phase I clinical trial, the vaccine was generally tolerable to immunized participants, with significant increase in hemagglutination inhibition and microneutralization titers (166). These findings were corroborated by a phase II viral challenge trial, highlighting that the vaccine is effective in inducing mucosal and humoral responses, with comparable efficacy to a commercially available quadrivalent vaccine against influenza A H1N1 (165). The study also highlighted the advantages of oral vaccination, including induction of mucosal immune response, maximal immunogenicity within the intestines, high uptake, and less invasive administration. An extended analysis of the trial showed that the oral vaccine also induced significant cellular immune responses, indicating potential protection from influenza viral shedding (167). Overall, the results of the trials were considered a success and more studies are expected to be done (https://investors.vaxart.com/, accessed on March 23, 2022). A vaccine similar to VXA-A1.1 is Ad4-H5-Vtn, another oral vaccine composed of an adenoviral vector expressing a recombinant HA. Unlike VXA-A1.1, Ad4-H5-Vtn uses an adenovirus type 4 vector, is non-adjuvanted, and is replication competent. The vaccine is produced by expressing H5 proteins in A549 cells through a BJ5183 bacterial vector (168). In a phase I trial, researchers found that after boosting with an inactivated H5N1 vaccine, the vaccine induced significant hemagglutination inhibition and seroconversion without severe adverse reactions (169). In another trial, researchers found that intranasal vaccine administration resulted in more robust cellular and humoral immunities with longer potency than oral administration (170). A more recent follow-up study indicated increased T-cell response and neutralizing antibodies against H5 persisting for 26 weeks post-immunization (171). Immunity was further increased after boosting with other commercially available RIVs and SVVs. The vaccine is sponsored by the National Institutes of Health and there are still no insights on future licensing.

Finally, a number of HA-based influenza mRNA vaccines are currently in their early phase clinical trials. Among the early candidates are VAL-506440 (mRNA-1440) and VAL-339851 (mRNA-1851) (Moderna, USA) which target influenza A/H10N8 and A/H7N9, respectively. Both vaccines are nucleoside-modified mRNAs in lipid nanoparticles (121). In their independent phase 1, randomized, double-blind, placebo-controlled trials, both vaccines had favorable safety profiles and induced robust humoral immune response but did not induce significant cellular response (172). Currently, both vaccines are not in the official product pipeline of Moderna (https://www.modernatx.com/research/product-pipeline, accessed 14 April 2022). Among the frontrunner influenza mRNA vaccines of Moderna is mRNA-1010, which is currently undergoing its phase I/II clinical trial. Unlike the previously described monovalent mRNA vaccines, mRNA-1010 is quadrivalent, targeting influenza A/H1N1, H3N1, B/Yamagata and B/Victoria (173). In the interim analysis of its clinical trial, the vaccine was found to induce non-superior humoral immune response and apparently higher reactogenicity compared to currently available influenza vaccines (174). More data is still needed before accurate insights about the vaccine can be made. Meanwhile, three other mRNA vaccines, MRT-5400, MRT-5401 (Sanofi, France/ Translate Bio, USA), and PF-07252220 (Pfizer, USA/ BioNTech, Germany), are currently undergoing phase I clinical trials. Unfortunately, the statuses of the first two are not readily accessible due to non-registration in clinicaltrials.gov. Both MRT-5400 and MRT-5401 vaccines are monovalent, targets influenza A/H3N2 (120), and are based on complexation with lipid nanoparticles (https://www.genengnews.com/news/sanofi-pivots-mrna-vaccine-program-from-covid-19-to-flu-pathogens/, accessed on April 14, 2022). PF-07252220 is based on modified RNA targeting influenza A/H1N1 and B/Yamagata in monovalent, bivalent and quadrivalent formulations (120). Among the primary outcomes of its ongoing trial include safety, reactogenicity, and HAI seroconversion rates. Other HA-based influenza mRNA vaccines are undergoing pre-clinical assessment; hence we expect more candidates under this vaccine type in the next few years.

#### HA Stalk-Based Recombinant Vaccines

Although the HA head is still the current standard target in influenza vaccine development, the HA stalk remains a promising option in designing universal vaccines. This is because the HA stalk is more conserved (inducing heterosubtypic immune response) and evolves slower than the HA head (reducing the need for regular updating of strain targets) (175). Supporting these notions are reports of broadly neutralizing antibodies toward the HA stalk, as summarized by Nath Neerukonda et al. (176). Interestingly, Nachbagauer et al. found that a recombinant HA stalk vaccine produced higher titers of broadly neutralizing antibodies in an age-dependent fashion (177). They hypothesized that recombinant HA vaccines produced in non-mammalian or avian cells have smaller glycans and a more accessible stalk domain since the steric hindrance in HA is minimized. Earlier studies also indicated that less glycosylated HA induces the production of cross-reactive HA stalk antibodies with increased receptor binding (178). Several studies have proposed different ways to develop HA stalk-based RIVs, as summarized by Bullard and Weaver (175).

A widely studied strategy in developing HA stalk-based RIVs is the creation of a chimeric HA (cHA). This is done by grafting HA head domains from different influenza subtypes to a conserved HA stalk. Immunization with a cHA vaccine requires a prime-boost approach, wherein each administration uses cHA with a different HA head but the same HA stalk (179). This approach works by allowing the immune system to produce HA head-specific antibodies (focus of the humoral response) and minute levels of conserved stalk antibodies during the primary dose. In the booster dose, researchers hypothesized that the immune system will refocus antibody production toward the conserved HA stalk since the HA head is antigenically different from that in the primary dose (175). cH8/1N1 is a recombinant cHA vaccine that recently concluded its phase I clinical trial. For the trial, the primary dose is composed of a LAV expressing an H8 head grafted to an H1 stalk and an N1 subunit from influenza A. The booster dose is composed of an inactivated SVV expressing an H5 head with the same H1 stalk and N1 subunit (180). The recombinant proteins were produced using baculovirus in High Five™ cells, while the SVV was produced in EB66 cells (181). The vaccine is administered either in a non-adjuvanted form, or adjuvanted with AS03, with the primary dose routed intranasally and the booster intramuscularly after 85 days (180). Results indicated that the adjuvanted form of the vaccine induced heterologous humoral response, with significant antibodies produced against H2, H9, H18, and H3 after the booster dose (181). The humoral immune response lasted 420 days after the primary dose. The antibodies produced against the vaccine also induced a strong and functional activity against the HA stalk, and detectable activity against conserved regions of the HA head. Finally, the vaccine also had acceptable safety, encouraging phase II clinical trials.

Another strategy to develop HA stalk-based vaccines is through production of a headless HA (hHA). The goal is to overcome HA head immunodominance and redirect the immune response toward the more conserved HA stalk (179). Traditionally, hHA is produced by treating HA with acidic and highly reducing agents to remove the HA1 subunit (comprising the whole globular head and some stalk domains) and expose the HA2 subunit (comprising most of the stalk domain) (182). Unfortunately, chemical treatment causes destabilization of the HA2 subunit, leading to conformational changes in its epitope (175). This can reduce the production of HA stalk antibodies, which is not ideal for vaccine development. Recent advancements involve expression of HA2 fragments in bacteria (183, 184), insect (185), and mammalian cells (186, 187) but cell-free protein synthesis has also been proposed (188). Among promising hHA-based vaccines, only VRCFLUNPF099-00-VP underwent a phase I clinical trial (NCT03814720). VRCFLUNPF099-00-VP is composed of an HA stalk from influenza A fused with *H. pylori* ferritin (clinicaltrials.gov, accessed November 10, 2021). Production was achieved using a structure-based strategy consisting of the removal of the HA head, thereby preventing any conformational change in the epitopes. The incorporation of bacterial ferritin allowed the production of a self-assembling HA stalk nanoparticle without inducing additional or autologous immune response (187). The purified vaccine contains eight HA trimers resembling HA stalk with typical epitope configuration. The recombinant proteins are expressed using lentiviral vectors in 293F human embryonal cells (189). Although its phase I clinical has been completed in 2021, no results have been officially published.

Other strategies for HA stalk-based recombinant vaccine production are currently being explored pre-clinically. These strategies include the following (1): creation of mosaic HA targeting both the HA stalk and conserved domains of the HA head for either influenza A (190) or B (191) (2); expression of a specific long alpha helix from the stalk regions of H3 (46) and H5 (192); and (3) hyperglycosylation of the HA head to mask its immunodominant epitopes and redirect the immune response toward the HA stalk (193). More studies on their efficacy to induce *in vivo* immune response are needed before clinical trials can be done on their safety and immunogenicity in humans.

### NA-Based Recombinant Vaccines

NA is the second major surface antigen of influenza, distributed at a ratio of 1:4 with HA (28). It undergoes slower antigenic evolution than HA (194), is thermostable (195), and broadly immunogenic depending on the delivery platforms (194, 196). These characteristics show the promise of NA in universal influenza vaccine development. Unfortunately, current seasonal vaccines lack standardization and regulations on NA dosage due to unestablished vaccine endpoints and activity markers (109, 151). Quantification of NA concentrations across the commercial inactivated vaccines Fluzone^®^, Fluvirin^®^, FluLaval^®^, and Flucelvax^®^ showed drastic differences ranging from 0.02 µg to 10.5 µg per dose (100). Nonetheless, Desheva et al. showed the presence of NA-inhibiting antibodies in human sera after vaccination with seasonal LAVs in clinical trials, suggesting their contribution to immunogenicity (197). The independence of NA immunity from HA has been demonstrated with influenza A pandemic strain H3N2, wherein past influenza infection with H2N2 contributed to NA-specific serological protection and infection reduction among individuals (198). In another example, researchers showed greater and continuous antigenic drifts in H1 and H3 influenza HAs, while N1 and N2 NAs were observed to undergo arrested drifts (199). NA antibodies are often not correlated with neutralizing activity and protection from primary infection, but instead disrupt NA activity, preventing the release of viral progeny and consequently reducing viral replication and disease severity (200).

NA immunogenicity depends on several factors such as the presence of other antigens, protein amount, structure and form, enzyme stability and activity, and vaccine delivery platforms. The association of HA and NA, as seen in seasonal vaccines, can cause low immune response toward NA due to antigenic competition and HA immunodominance (190). This problem can be circumvented through lengthening of the NA stalk (194) or exchanging the 5’ and 3’ terminals of the HA and NA gene segments (201). The number of NA tetramers determines the ability of vaccines to induce NA antibody production and corresponding immunogenicity (202). Immunization with purified recombinant N1 or N2 in their tetrameric forms protected mice from lethal challenge with the homologous influenza strains H1N1 and H5N1 or H3N2, respectively (203–205). Crucial to the formation of tetrameric structures are cysteine residues along the NA stalk. The introduction of cysteines along the NA stalk were found to generate recombinant NAs with enhanced enzymatic activities, protection of mice from weight loss and mortality, and higher NA-inhibiting antibody titers in mice (206). NA dose dependency in recombinant protein, DNA, or VLP vaccines has also been demonstrated, wherein higher concentrations relate to increased NA inhibition titers, antibody-secreting B cells, IgG titers, and survival rates (109, 207). Menne et al. observed that 10 and 15 µg of the subtype N2 in VLPs elicited similar antibody titers (109). The NA administration pathway can also affect vaccine immunogenicity depending on the delivery platform. For recombinant NA proteins, intranasal administration has been observed to elicit better cellular and humoral immunity than the intramuscular route, probably due to IgA antibodies produced, that can recognize more antigenic epitopes than NA-specific IgGs (204, 208). In VLPs, intramuscular administration of N2 has been shown to completely protect mice from heterologous viral challenge over intranasal delivery (109). Live vector vaccines, such as NA-expressing lactic acid bacteria, can also induce humoral and mucosal responses upon oral administration in chickens (116).

In terms of stability, Sultana et al. evaluated the enzyme activities of NA components in inactivated influenza vaccines produced for the 2011/2012 influenza seasons, after treatment with heat, detergent, and freeze–thawing (209). They found that the strain, composition, and shelf-life affected enzyme stability and immunogenicity. Similarly, the presence of cations, such as calcium and magnesium, preserved recombinant NA vaccine activity under low temperatures, but did not affect immunogenicity and protection in mice (195). The study also demonstrated that vaccine stability and activity were similar when stored at 4°C and −80°C for at least eight months, indicating flexibility in vaccine storage.

Different NA-based recombinant vaccine platforms can offer their own advantages and drawbacks. Recombinant NA proteins are well regarded for their safety but have limited immunogenicity. While mice vaccinated with recombinant N1 or N2 NA proteins were protected against homologous and partially against heterologous viral challenges, the combination of recombinant N3–9 NA proteins failed to protect against H1N1 and H3N2 lethal challenges, suggesting the absence of heterosubtypic protection (100). However, in the same study, NAs from influenza B (Yamagata lineage) were found to provide protection against two influenza B Victoria lineages with antigenically distinct HAs. Similarly, Deng et al. also observed this subtype-specific immunity wherein immunization with recombinant NA protein from the subtype H7N9 provided heterosubtypic protection against H1N1 in mice, while homologous H1N1 and heterologous H5N1 NA immunizations did not (203). Both H1N1 and H5N1 recombinant NA proteins also failed to provide protection against heterosubtypic H7N9 regardless of dosage (207). Despite these limitations, cluster-based consensus approach, combining NA amino acid sequences of various influenza strains, can also be used to broaden protection of recombinant NA protein vaccines (205). NA-based DNA vaccines have also shown immunogenicity comparable, or in some cases better than, HA-based platforms. In a single-dose set-up, plasmid DNA encoding NA from H1N1 administered through electroporation in mice led to 100% survival against homologous challenge and protection for neonatal mice, while HA DNA immunizations led to little to no survival (210). Immunizing twice with HA or NA DNA from avian influenza H9N2 showed homologous protection with 100% survivability even at just a 3-µg dosage. NA DNA vaccines can also be co-expressed with other antigens such as HA, NP, M1, and M2 to expand immunity against homologous and heterologous infections as an alternative to purified recombinant protein-based combinations, as observed in mice, pig, and ferret models (211–213). However, heterosubtypic immunity for recombinant NA protein and DNA-based vaccines remain lacking. In contrast, VLP-based NA-recombinant vaccines have shown to induce a heterosubtypic level of protection. Mice vaccinated with H1N1 NA in M1 VLPs were protected against lethal challenges of homologous H1N1 and heterosubtypic H3N2 influenza, and induced Th2-based IgG1 antibody production (196). In another study, H1N1 NA VLPs induced predominantly IgG2a antibodies and provided cross-protection against heterologous H5N1 and heterosubtypic H3N2 following intranasal challenge in mice (194). Some subtypes of NA such as N2 failed to induce heterosubtypic immunity in VLP platforms (109, 151). However, multivalent VLPs containing NA from H1N1 and H3N2 subtypes can be used to induce a substantial IgG response and protect against both influenza subtypes without antigenic competition (109).

Another option to increase NA immunogenicity are vector-based vaccines. Recombinant *Lactococcus lactis* expressing NA from H5N1 provided complete protection against homologous challenge in chickens *via* humoral and mucosal responses with high levels of NA-specific IgG and IgA antibodies, even without adjuvants (116). NA in live viral vectors also showed comparable immunogenicity to HA. In nonhuman primate models, attenuated Newcastle disease virus vector carrying HA or NA from H5N1 administered through intranasal and intratracheal routes induced high levels of neutralizing antibodies upon a second dose. This may be due to affinity maturation and facilitated protection against homologous and heterologous H5N1 influenza strains (214). Similarly, MVA vectors carrying the avian influenza H7N3 or H7N9 HA or NA genes in mice induced NA-specific antibodies with some level of cross-protection (215). Co-expression of both antigens in this vector did not show inhibition of immune reactions. Hence, NA shows great potential in eliciting homologous to heterosubtypic immunity depending on the recombinant platforms. However, there is still a need to standardize and regulate its dosage as well as determine endpoints prior to developing vaccines for clinical trials.

### M1- and NP-Based Recombinant Vaccines

Expanding the antigenic repertoire is key to combat emerging influenza pandemics. Internal influenza proteins such as M1 and NP can also induce cross-reactive immunity (216). In an *in silico* study of influenza A strains, several highly conserved regions were detected in the PB1, PB2, PA, NP, M, and NS genes (217). They also induced strong CD4^+^ and CD8^+^ T-cell responses, which enhanced antibody production for heterosubtypic immunity (218, 219). Their conservation, immunogenicity, and complementary protection make them attractive targets for universal influenza vaccine production. However, like NA, these antigens remain to be standardized. Three licensed trivalent inactivated influenza vaccines from 2007–2008 in the USA (Fluzone^®^, Flulaval^®^, and Fluvirin^®^) induced different M1- and NP-specific cytotoxic T-cell responses *in vitro* and accordingly different M1 and NP protein levels detected (220). Although M and NP antibodies are often not correlated with neutralizing activity (219, 221), they bind to highly conserved antigenic epitopes, reduce viral propagation and disease, and involve antibody-dependent phagocytosis and cytotoxicity (222–224). They can also activate natural killer cells (225).

In mice, intranasal administration of purified recombinant M1 protein from avian influenza H9N2 showed dose-dependent complete protection against lethal H9N2 and to some extent, against heterologous challenges of H1N1(70%) and H5N1 (30%) (226). Protection was further improved by adding chitosan adjuvants inducing both systemic IgGs and secretory IgAs. Oral administration of M1-based DNA vaccines enclosed in cationic liposomes induced M1-specific IgGs, IgAs, cytotoxic T cells, and cytokine production upon homologous challenge (227). Prime-boost approach with these components extended immunogenicity. Intranasal priming of mice with recombinant M1 DNA and boost with M1 protein from H9N2 elicited dose-dependent immune responses with complete protection against homologous H9N2 and partially against heterosubtypic H1N1 challenges (228). M1 alone may not be sufficiently immunogenic, so it is often used to generate VLPs with other antigens such as HA, NA, and M2 to provide better homologous to heterosubtypic immunity against influenza A and B (196, 229–234). Similarly, vaccines from recombinant DNA and vaccinia vector, or prime-boost using these platforms with M1 from H3N2, induced low humoral (IgG) and cellular (IFN-γ) immune responses and low protection against heterologous H1N1 challenge. M1 combination with other internal antigens such as NP and PB1 showed higher immune response and full protection (117). The fusion of M1 and HA2 (HA monomer subunit) in the *L. lactis* vector for oral vaccination in chickens induced antibodies, cytokines, and T-cell mediated immune response with comparable protective efficacy to that of H9N2 WVVs (235). However, M1 VLPs using adenoviral dodecahedron structures were able to efficiently enter and activate myeloid dendritic cells, facilitate antigen presentation, and induce CD8^+^ T cells (236). Hence, these studies suggest that M1 mostly serves as a complement to other antigens to enhance vaccine uptake and conferred protection.

In contrast with M1, NP showed extensive immunogenicity across different platforms. NP recombinant DNA, vaccinia vector, and prime-boost of both NP recombinant DNA and vaccinia vector from H3N2 induced the strongest humoral and cellular responses, with full protection against H1N1 (117). In another study, a single dose of NP DNA alone or with M1 DNA from H5N1 provided partial protection against homologous challenge in H1N1 pre-exposed mice (237). NP heterosubtypic protection can also depend on antibodies (222, 238) and can last for a period of one year or more (239). Boosting of NP-reactive antibodies using NP protein or IgGs has been shown to enhance heterosubtypic immunity and accelerate viral clearance and protection against lethal H1N1 and H3N2 challenges (240). Intranasal immunization of mice with NP recombinant protein from H1N1 and compound 48/80 adjuvant provided complete protection against lethal homologous H1N1 and decent protection against heterologous (H5N1) and heterosubtypic (H9N2) challenges (99, 101) Similarly, intramuscular immunization with NP from H1N1 with SLA-SE or alhydrogel adjuvants protected young mice against homologous lethal challenge (241).

NP immunogenicity can also be enhanced through VLPs and protein oligomerization. Savard et al. developed papaya mosaic virus capsid VLPs with innate adjuvant properties to carry the NP from H1N1 that induced improved immune responses, viral clearance, and recovery against homologous challenge in mice (242). NPs from influenza B Yamagata and Victoria lineages placed in adenoviral vectors were also able to induce similar NP-specific IgG and CD8^+^ responses, and complete cross-protection to challenges of both lineages in mice (243), which suggest expansive immunity conferred by recombinant NP-based vaccines. OVX386, a novel recombinant vaccine based on oligomerized NP proteins developed by Del Campo et al. has been shown to induce improved IgG, CD4^+^ and CD8^+^ T-cell responses, uptake of NP antigens into dendritic cells, broad protection against H1N1 and H3N2 lethal challenges in mice and can be used with inactivated influenza vaccines to increase protection (244). OVX836 vaccination also showed strong long-lasting cross-protection against lethal influenza challenges for a minimum of 90 days in mice (245). Recently, a phase I clinical trial of OVX836 in adults showed no adverse events across administrations or dosage levels. The study also showed increased NP-specific IFN-γ T cells and IgG titers at first dose, however, immune response was not further increased at the second dose (246). Overall, NP-based recombinant vaccines showed potential as a main or component antigen in inducing CD8^+^ T-cell responses crucial for heterosubtypic immunity in universal influenza vaccine development.

Combinations of M1 and NP are also promising candidates. In DNA vaccines, combination of both from H5N1 conferred improved protection against lethal homologous and heterologous (H1N1) challenges (247). Several animal studies and human clinical trials (phase I/II) have also demonstrated that MVA or adenoviral vectors containing recombinant M1 and NP induce strong and long-lasting homologous to heterosubtypic immunity against influenza, with little adverse events even in elderly populations (218, 248–253). Replication-deficient chimpanzee adenoviral vectors (ChAdOx) such as ChAdOx1 serotype carrying NP and M1 have also been shown in phase I clinical trials to be safe and immunogenic, with long-term broad protection when coupled with MVA vectors carrying the same antigens in heterologous prime/boost regimens (254, 255). More recently, using another ChAdOx serotype (ChAdOx2) containing NP, M1 and NA, T-cell and antibody immune responses increased upon aerosol delivery in H1N1 pre-exposed pigs (256). Meanwhile, Flu-v, developed by PepTcell (SEEK), contain synthetic NP, M1 and M2 influenza protein antigens. While its phase I and II clinical trials showed safety, dose-dependent immune response, and protection against H1N1 and H3N2 challenges, additional studies are needed to evaluate induction of cellular immunity (257–259). M1 and NP in MVA combined with the HA stem, PB1 and M2 from H5N1, H7N1, H9N2, and H1N1 subtypes were shown to protect against a wide range of influenza strains and induced specific antibodies and CD4^+^ and CD8^+^ T-cell responses (260). Similarly, addition of NP in VLPs with antigens such as HA, NA, and M1 enhanced humoral and cellular responses, and induced broad protection against heterologous viral challenges in chickens (261). Yang et al. constructed recombinant H1N1 NP and M1 proteins with HSP60, and then immunized mice with an oil-in-water adjuvant through the intranasal route. This combination induced balanced IgG1 and IgG2a levels, high mucosal and cellular responses, and complete protection against lethal H7N9 challenge (262). In a pre-print article, prime immunization with recombinant H1N1 NP and M1 DNA vaccine containing calreticulin, another HSP, followed by boosting with live attenuated influenza vaccine in mice was shown to confer better protection than commercial SVV against lethal H1N1 challenge (263). A novel delivery system using self-amplifying mRNA carrying M1 and NP also demonstrated robust IgG, CD4^+^ and CD8^+^ T-cell responses for NP, and protection against homologous H1N1 and heterosubtypic H3N2 infection in mice (264). Taken together, M1 and NP formulations are safe and immunogenic, and can be coupled with other antigens or co-administered with other vaccines for a broader range of protection.

### M2-Based Recombinant Vaccines

Influenza matrix proteins have been shown to evolve independently (265). M2 can induce antibodies independently of natural killer-mediated response and bind to specific M2 epitopes (266). The ectodomain (M2e) of M2 provides some immunogenicity depending on the platform used (267). M2 or M2e alone often has poor immunogenicity. Low and short-lived M2e-specific antibody responses were observed in influenza-infected mice, and suboptimal or absent in naturally-infected humans (268). Nonetheless, several strategies can be employed to improve recombinant M2e immunogenicity and broaden protection up to heterosubtypic levels. These include fusion with antigenic proteins such as tetrameric rotavirus protein fragments (269), *Mycobacterium tuberculosis* HSP70 protein (270), cholera toxin subunit or *Staphylococcus aureus* protein A (271), and conjugation with hemocyanin or outer membrane protein complex of *Neisseria meningitidis* (272). In mice and rabbits, fusion of M2e with *Salmonella* flagellin proteins which are TLR5 ligands, induced superior IgG responses despite low dosage than conventional M2e with an alum adjuvant, as well as protection against lethal homologous challenge, regardless of the administration route, suggesting diverse immune response mechanisms (273). Double-blinded, randomized, placebo-controlled phase I and II clinical trials with this formulation also showed high safety and induced high M2e-specific antibodies, especially when combined with trivalent inactivated vaccines (274, 275). In DNA vaccine platforms, M2 can be generated with other antigens to enhance the potency and immunogenicity spectrum. M2 with HA stems or HA consensus genes and cytotoxic T-cell epitopes demonstrated efficient immune induction, with some level of cross-protection against lethal challenges (276, 277). Park et al. demonstrated complete heterosubtypic protection in mice against H5N2 after administration of a DNA vaccine containing M2e and H1 (104). These studies suggest that recombinant M2-based protein and DNA vaccines should be administered with other antigens to evoke sufficient immune responses.

M2 or M2e in VLPs are also viable vaccine candidates. Intranasal or intramuscular prime-boost administration of VLPs containing M2e tandem repeats of influenza from different host species conferred heterosubtypic immunity with effective IgG2a (Th1 cells response), CD4^+^ and CD8^+^ T-cell responses, and superiority over commercial influenza SVVs and HA-based VLPs (278–281). Conversely, supplementation of commercial influenza SVVs with M2e repeats in VLPs can extend specific immunity to long-lasting cross-protection against heterosubtypic viral challenges (282). VLPs containing M2e tandem repeats coated in microneedle patches, a minimally invasive and painless delivery system, showed epitope stability and remained immunogenic at room temperature for eight weeks (283). The vaccine also induced IgG2a, M2e-specific antibodies, and IFN-γ T-cell responses with protection against heterosubtypic viral challenges comparable or better than intramuscular delivery in mice (283). Combinations of M2e with other antigens in VLPs have also been explored. M2e with flagellin in VLPs or co-administration of M2e and flagellin VLPs in mice elicited higher levels of IgGs and IgAs, activated M2e-specific T-cell responses, and provided better protection against homologous and heterosubtypic challenges than M2e alone, in VLPs or with adjuvants (284, 285). M2e can also be carried in hepatitis B core (HBc) VLPs with NP and oil-in-water adjuvant to stimulate IgG1 (Th1) and IgG2a (Th2) antibodies, IFN-γ, and cross-protection against lethal H1N1 and H5N1 challenges in mice (286). Thus, there is flexibility in M2-based VLP platforms for enhancement of immune response despite the natural low immunogenicity of the antigen alone. Various vectors can be used to deliver M2-based antigens or VLPs. Ameiss et al. used HBc VLPs containing M2e common to avian influenza and transformed attenuated *Salmonella* Typhimurium with delayed lysis phenotype for oral administration in mice (114). Their study showed increased IgG2a and IgA levels and moderate protection against avian influenza than for non-lysis *Salmonella* phenotype. In contrast, ACAM FLU-A by Sanofi Pasteur is a recombinant M2e-based vaccine produced using HBc. Its phase 1 clinical trial indicated considerable safety and immunogenicity. However, no further updates from the manufacturer have been published since 2012, most probably due to waning antibody titers of the vaccine even with adjuvants (NCT00819013; clinicaltrials.gov, accessed on March 31, 2022). Several studies have demonstrated the utility of different viral vectors, including MVA, adenovirus, tobacco mosaic virus, human papillomavirus, and norovirus, in presenting M2e. They induced significant, long-lasting, and broad humoral cellular and mucosal responses, which can be further improved when M2e is co-expressed with other antigens such as NP (115, 282, 287–289). Therefore, despite its overall lower immunogenicity, M2 has the potential to play antigenic roles in recombinant vaccine development if administered as tandem repeats in VLP platforms. M2 can also be utilized in combination with other antigens or vaccine formulations and in different administration routes to enhance immune efficacy and duration.

### PA-, PB1-, and PB2-Based Recombinant Vaccines

Few studies on polymerase complex subunit-based RIVs have been conducted. Nonetheless, these internal antigens have several indirect utilities in vaccine development. Through plasmid-based reverse genetics, the proteins are used to regenerate RNA genomes from plasmid complementary DNA (viral rescue) during construction of RIVs in transfected cell lines (290, 291). They also contribute to temperature sensitivity caused by mutations in PA, PB1, or PB2 in recombinant live attenuated avian influenza vaccine strains, which conferred protection in chickens (292, 293). Addition of an HA epitope tag to the PB1 C-terminal of these mutants further attenuated vaccine strains and provided immunity against homologous and heterologous challenges in mice (294). In influenza B, PA, and PB2 can also independently contribute to temperature sensitivity (295). PB1 gene sources have been shown to affect HA yield and growth of influenza vaccine strains (296). In addition, PB1 mutation can be used to generate high fidelity and genetically stable recombinant live LAVs (297, 298).

Differences in immune induction have also been documented across influenza polymerase antigens. Vaccination in mice with PA protein through peptide-pulsed dendritic cells showed poor and delayed viral clearance in contrast with NP, but still induced CD8^+^ and cytotoxic T-cell responses (299). In a DNA vaccine platform, while both PB1 and PB2 plasmids from H1N1 and H3N2 induced Th1-biased immune responses, only PB1 conferred protection against homologous and heterologous challenges (300). However, in another study, PB1 from H3N2 delivered through DNA, recombinant vaccinia virus vector, or prime-boost of both provided weak protection against H1N1 challenge in mice, but conferred complete protection when combined with NP and M1 (117). PB1 immunogenicity can be improved by linking it to a murine invariant chain protein which is involved in antigen presentation and functions as a chaperone to major histocompatibility complex class II. The improved antigen showed increased CD8^+^ T-cell responses and long-lasting partial protection in mice when encoded in an adenoviral vector administered locally and systemically (118). Unfortunately, the induced immune response is still inferior to NP vaccination, probably due to the less stable expression of PB1 on cell surfaces.

Combinations with other antigens have also been explored. A synthetic long peptide vaccine comprised of influenza epitopes for B- (HA2 and M2e) and/or T cells (NP, PB1, and M1) stimulated IgG and IFN-γ production in mice and ferrets (301). However, despite some reduction in viral titers and disease severity, the vaccines provided little to no protection against sublethal challenges. In contrast, Ichihashi et al. showed that vaccination of mice with single influenza epitopes (e.g., from PA, PB1, PB2, and M2) targeting cytotoxic T-cell response provided limited protection with varying immunogenicity (302). Combining specific epitopes with intranasal administration offered complete protection against lethal H5N1, H1N1, and H3N2 challenges. Xie et al. described that consensus internal antigen (PA+PB1+M1 and NP+PB2+M2) vaccines administered intramuscularly *via* prime immunization with DNA and boosting with adenoviral vector carrying consensus antigens showed higher IFN-γ production to specific antigens than DNA priming and vaccinia vector boosting, which showed a low but broader cellular response to different antigens (303). In viral challenge studies, DNA and vaccinia vector vaccines also conferred better protection than adenoviral vectors against H1N1, although neither protected against H7N9 lethal challenges. However, intranasal administration provided cross-protection against these challenges, especially with DNA priming and adenoviral vector boosting. Collectively, polymerase-based RIVs may induce an immune response in combination with other antigens. However, they require further research to evaluate immunogenicity, efficacy, and safety as vaccines.

### Deantigenized Recombinant Vaccine

**Figure 5:**
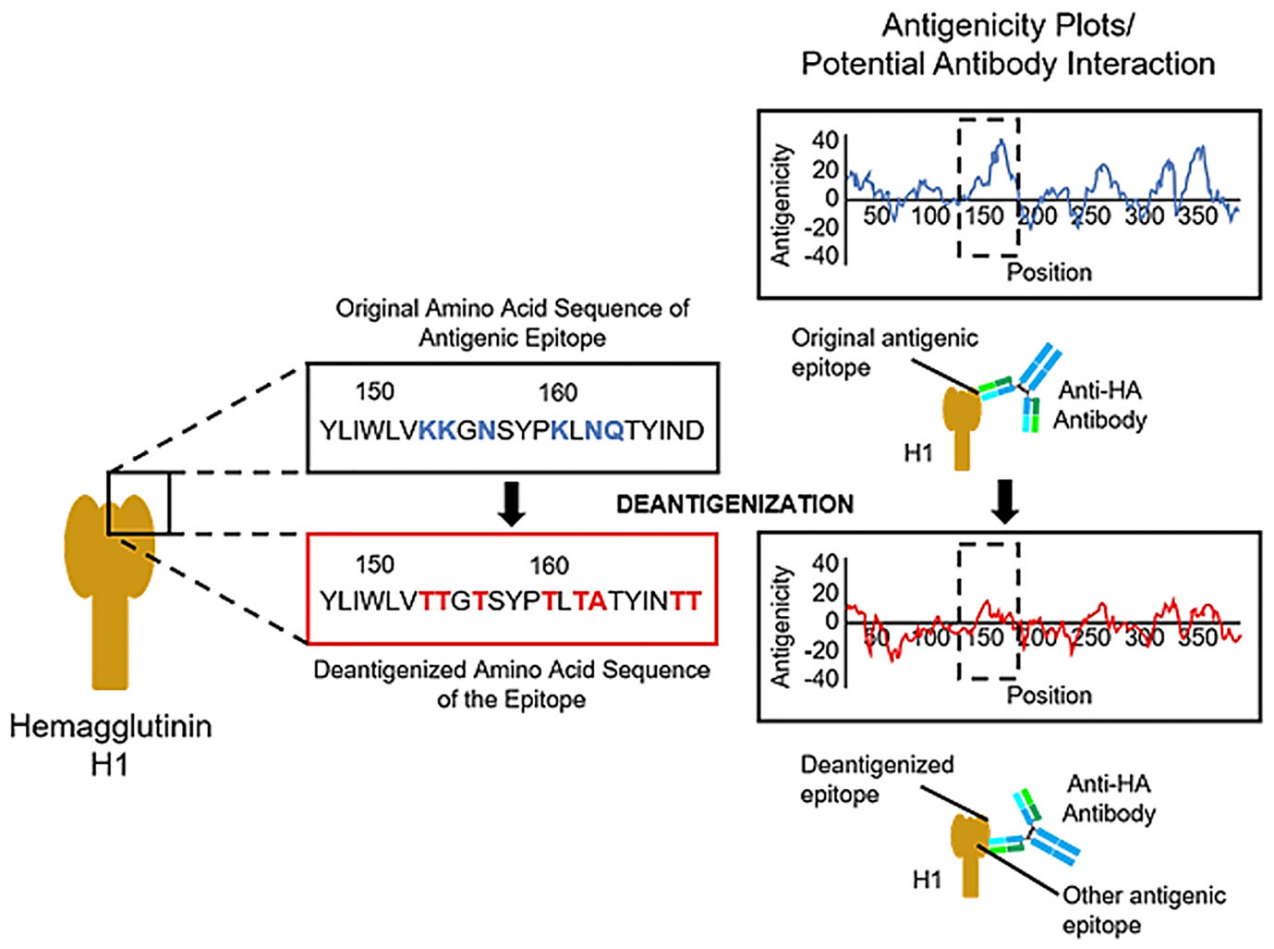
Theoretical representation of the deantigenization of the influenza H1 protein with potential application in vaccine development. Deantigenization can be done on specific sites of the target protein that exhibit high solvent accessibility and antigenicity (based on calculated values), in this case an area near the receptor-binding site of the H1 protein. Amino acid substitution was introduced in this region following the replacement rule described by Padlan (305), particularly the change of Ks, Ns, and Qs to mostly Ts given the sheet configuration of the peptide in the area. The strict ruling on amino acid replacement renders the site less antigenic (within the broken line box in the antigenicity plot) without changing the overall three-dimensional configuration of the protein. By deantigenizing highly antigenic epitopes of the protein, other antigenic sites can be exposed or targeted by antigen-recognizing cells, potentially leading to the production of antibodies targeting other more conserved epitopes of the H1.

With constant antigenic evolution among influenza viruses, researchers proposed deantigenization as a novel strategy to enhance vaccine activity. Deantigenization involves explicit manipulation of targets’ antigenic properties. Specifically, amino acid sequences of immunodominant sites of a target molecule (e.g., HA head) are modified to lower their antigenicity without changing the overall protein configuration (304). By “deantigenizing” the immunodominant sites, researchers hypothesized that the immune system will divert antibody production toward other antigenic sites of the protein, such as conserved regions of the molecule that may be masked by the immunodominant epitope (**Figure 5**; 305). This strategy may result in a longer-lasting immune activity against influenza viruses, regardless of the strain (306). Deantigenization is synonymous to the previously described deceptive imprinting and immune refocusing processes in vaccine design (307, 308).

Previous immunomodulating strategies for antigens include immunodominant epitope excision, glycosylation, and direct amino acid replacement. In deantigenization process described herein, amino acid replacement considers the native configuration of the antigen together with purposeful focusing of antigenicity toward other antigenic sites (305). The overall steps of deantigenization of target epitopes involve the (1) identification of the target protein (i.e., preferably one that elicits a strong immune response) and its active sites; (2) mapping of the three-dimensional structure of the protein and all its epitopes; (3) identification of accessible antigenic sites of the protein aside from active sites and known epitopes; and (4) replacement of amino acid residues of the accessible antigenic sites using strict guidelines to reduce the immunogenicity of the target while maintaining its three-dimensional configuration and at least one Th-cell epitope (305). Mikita and Padlan successfully used this process to produce, *in silico*, a deantigenized HA antigen with increased antigenicity toward a putative HA cleavage site that is usually shielded by the HA head (309). The researchers proposed the use of the deantigenized amino acid sequence to develop a recombinant, cell-expressed subunit vaccine against seasonal influenza. Their extended analysis indicated that deantigenization also worked when targeting influenza B viruses, making the deantigenized HA effective to the B subtype, much like a multivalent vaccine with heterosubtypic activities (309). Despite the potential of deantigenization for producing universal vaccine candidates, no *in vivo* experimental research nor clinical trials have been conducted to confirm their hypothesized activities. However, in combination with more advanced protein analysis and fast recombination and protein expression systems, it is not impossible to see a rise in interest in the use of deantigenization for development of RIVs that may induce heterosubtypic immune responses.

## Future Directions

Several influenza antigens and strategies for developing RIVs have been discussed in this review, each offering different levels of immunogenicity and potential utility. While recombinant HA remains the most studied, with numerous candidates undergoing clinical trials, the antigen may exhibit high variability across influenza strains and immunodominance of its globular head. Hence, research is being conducted involving more conserved regions such as the HA stalk, combinations of HAs from different influenza subtypes, or formulations with more conserved influenza antigens and epitopes. Alternatively, NA, which evolves independently and slower than HA, has also been explored. However, NA remains to be standardized in terms of dosage and clinical endpoints in vaccine production, and clinical trials have yet to be conducted to evaluate their safety and efficacy. In summary, production of recombinant vaccines (based on either surface or internal influenza antigens) is a relevant approach to explore in influenza immunization. However, as more researchers propose advanced techniques in vaccine production, we can expect a shift in paradigm in vaccine development, from using whole viral particles, protein subunits, up to using viral genetic material. This shift can be seen in the current vaccine development for COVID-19, where mRNA vaccines are significant candidates in providing immunization against the infection (310). Recent progress using mRNA platforms has also been made for influenza vaccines, with a few HA-based candidates in early phases of clinical trials. While some influenza mRNA vaccines (VAL-506440 and VAL-339851 by Moderna, USA) stopped in early trials due to lack of immune response induction (121, 172), other candidates such as mRNA-1010 (Moderna, USA) showed significant potential but still requires further clinical trials to obtain more data about their safety and efficacy (174). In addition, there are also some candidates in pre-clinical stages (120, 172). In prospect, recombinant nucleic acid-based platforms can be viewed as a faster and safer production technique for influenza vaccines.

While surface antigens remain the most attractive vaccine targets, internal antigens have also shown promise due to their highly conserved epitopes. NP showed strong induction of the immune response, including heterosubtypic protection, across different recombinant platforms. Immunity can also be enhanced through addition of adjuvants or combination with other antigens such as PB1, PB2, and M1. Meanwhile, influenza matrix proteins are not as immunogenic alone and have been shown to require other influenza antigens, epitopes from other organisms, and different vaccine platforms to elicit a viable immune response. Internal antigens within the polymerase complex (PA, PB1, and PB2) have other utilities such as reverse genetics in recombinant vaccine production as well as in enhancing temperature sensitivity and genetic stability of recombinant LAVs. Other unique recombinant vaccine techniques have also been explored as with the case of DeltaFlu (Vivaldi Biosciences Inc., USA), an intranasal vaccine based on replication-deficient influenza virus with deleted NS1 gene. Phase I and II clinical trials of the vaccine showed heterosubtypic protection, inducing significant seroconversion with mild adverse events (311, 312). Finally, deantigenization of variable and immunodominant influenza epitopes to modulate the immune response toward more conserved sites can, theoretically, provide long-lasting and heterosubtypic protection. Further studies on this concept are recommended to improve universal vaccine candidates.

Aside from improving immunogenicity and the production process, RIV development should also consider cost-effectiveness and vaccination program implementation (313). Thus, assessment of vaccine development, manufacturing, and distribution capabilities, and increased vaccine research and clinical trials should be conducted to facilitate vaccine production and implementation in low- and middle-income countries. Finally, influenza has recently been reported to co-infect with SARS-CoV-2 with increased severity and mortality (314, 315). Therefore, vaccination strategies considering other viruses with pandemic potential should be pursued, expanding the concept of universal influenza vaccines to cover other infectious diseases. The COVID-19 pandemic also paved the way for other RIV development platforms such as mRNA-based vaccines.

In summary, the progress in RIVs provides a glimpse of the future of universal vaccine production, banking on the improved immunogenicity, production timeline, and novelty of RIV development. Therefore, efforts to enhance and fast-track the research in this field of vaccine production could potentially help in accelerating our goal toward universal influenza vaccination.

## Author Contributions

MC and RP conducted the review of literature and wrote the manuscript. WR reviewed, edited, and provided expert opinion on the manuscript. All authors conceptualized the review paper and approved the final version of the manuscript.

## Funding

This study was supported by the Emerging Inter-Disciplinary Research (EIDR) Grant of the Office of the Vice President for Academic Affairs of the University of the Philippines System (Project No. C04-003) and the DA-Biotechnology Program Office (Project No. DABIOTECH-R2007).

## Conflict of Interest

The authors declare that this review was conducted without any commercial or financial relationships that could be construed as a potential conflict of interest.

## Publisher’s Note

All claims expressed in this article are solely those of the authors and do not necessarily represent those of their affiliated organizations, or those of the publisher, the editors and the reviewers. Any product that may be evaluated in this article, or claim that may be made by its manufacturer, is not guaranteed or endorsed by the publisher.
