# Corrigendum: Recent Progress in Recombinant Influenza Vaccine Development Toward Heterosubtypic Immune Response

**DOI:** 10.3389/fimmu.2022.948031

**Published:** 2022-06-13

**Authors:** Mark B. Carascal, Rance Derrick N. Pavon, Windell L. Rivera

**Affiliations:** ^1^ Pathogen-Host-Environment Interactions Research Laboratory, Institute of Biology, College of Science, University of the Philippines Diliman, Quezon City, Philippines; ^2^ Clinical and Translational Research Institute, The Medical City, Pasig City, Philippines

**Keywords:** heterosubtypic immunity, influenza, influenza antigen, recombinant vaccine, universal vaccine

In the original article, there was a mistake in **Figure 4** as published. An old version of **Figure 4** was used instead of the latest figure file. The corrected **Figure 4** appears below.

**Figure 4:**
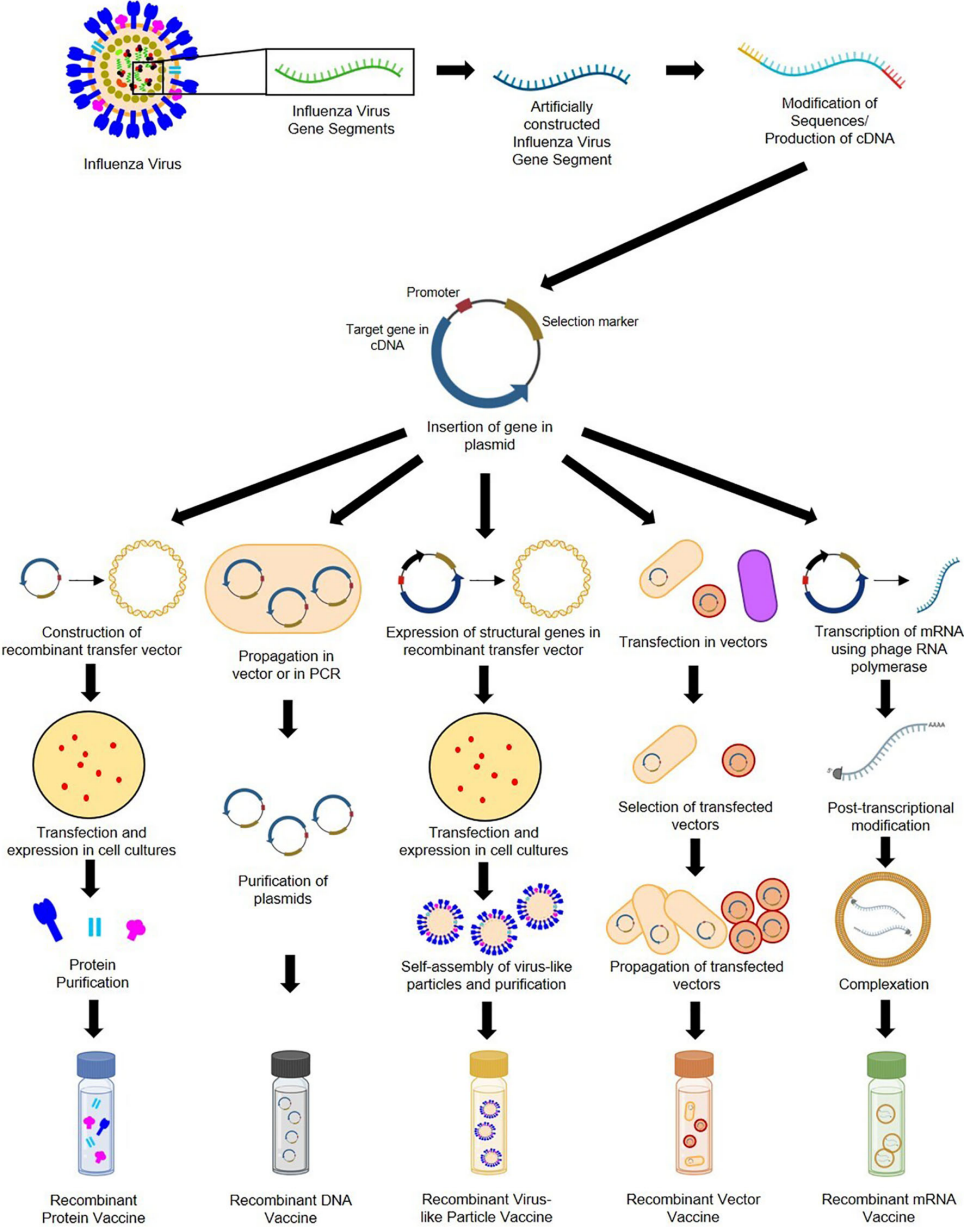
General construction process of recombinant protein, DNA, VLP, vector-based, and mRNA influenza vaccines.

The authors apologize for this error and state that this does not change the scientific conclusions of the article in any way. The original article has been updated.

## Publisher’s Note

All claims expressed in this article are solely those of the authors and do not necessarily represent those of their affiliated organizations, or those of the publisher, the editors and the reviewers. Any product that may be evaluated in this article, or claim that may be made by its manufacturer, is not guaranteed or endorsed by the publisher.

